# A YAP/TAZ-CD54 axis is required for CXCR2^−^CD44^−^ tumor-specific neutrophils to suppress gastric cancer

**DOI:** 10.1093/procel/pwac045

**Published:** 2022-10-26

**Authors:** Pingping Nie, Weihong Zhang, Yan Meng, Moubin Lin, Fenghua Guo, Hui Zhang, Zhenzhu Tong, Meng Wang, Fan Chen, Liwei An, Yang Tang, Yi Han, Ruixian Yu, Wenjia Wang, Yuanzhi Xu, Linxin Wei, Zhaocai Zhou, Shi Jiao

**Affiliations:** CAS Center for Excellence in Molecular Cell Science, Institute of Biochemistry and Cell Biology, Shanghai Institutes for Biological Sciences, Chinese Academy of Sciences, University of Chinese Academy of Sciences, Shanghai 200031, China; State Key Laboratory of Genetic Engineering, School of Life Sciences, Zhongshan Hospital, Fudan University, Shanghai 200438, China; Tongji University Cancer Center, Department of Medical Ultrasound, Shanghai Tenth People’s Hospital, Ultrasound Research and Education Institute, Tongji University School of Medicine, Shanghai 200072, China; Postdoctoral Station of Clinical Medicine, Shanghai Tenth People’s Hospital, Tongji University School of Medicine, Shanghai 200072, China; Tumor Immunology and Gene Therapy Center, Shanghai Eastern Hepatobiliary Surgery Hospital, Shanghai 200438, China; Department of General Surgery, Yangpu Hospital, Tongji University School of Medicine, Shanghai 200090, China; Department of General Surgery, Hua’shan Hospital, Fudan University Shanghai Medical College, Shanghai 200040, China; State Key Laboratory of Genetic Engineering, School of Life Sciences, Zhongshan Hospital, Fudan University, Shanghai 200438, China; CAS Center for Excellence in Molecular Cell Science, Institute of Biochemistry and Cell Biology, Shanghai Institutes for Biological Sciences, Chinese Academy of Sciences, University of Chinese Academy of Sciences, Shanghai 200031, China; State Key Laboratory of Genetic Engineering, School of Life Sciences, Zhongshan Hospital, Fudan University, Shanghai 200438, China; State Key Laboratory of Genetic Engineering, School of Life Sciences, Zhongshan Hospital, Fudan University, Shanghai 200438, China; CAS Center for Excellence in Molecular Cell Science, Institute of Biochemistry and Cell Biology, Shanghai Institutes for Biological Sciences, Chinese Academy of Sciences, University of Chinese Academy of Sciences, Shanghai 200031, China; State Key Laboratory of Genetic Engineering, School of Life Sciences, Zhongshan Hospital, Fudan University, Shanghai 200438, China; Tongji University Cancer Center, Department of Medical Ultrasound, Shanghai Tenth People’s Hospital, Ultrasound Research and Education Institute, Tongji University School of Medicine, Shanghai 200072, China; Tongji University Cancer Center, Department of Medical Ultrasound, Shanghai Tenth People’s Hospital, Ultrasound Research and Education Institute, Tongji University School of Medicine, Shanghai 200072, China; Tongji University Cancer Center, Department of Medical Ultrasound, Shanghai Tenth People’s Hospital, Ultrasound Research and Education Institute, Tongji University School of Medicine, Shanghai 200072, China; State Key Laboratory of Genetic Engineering, School of Life Sciences, Zhongshan Hospital, Fudan University, Shanghai 200438, China; State Key Laboratory of Genetic Engineering, School of Life Sciences, Zhongshan Hospital, Fudan University, Shanghai 200438, China; Department of Stomatology, Shanghai Tenth People’s Hospital, Tongji University School of Medicine, Shanghai 200072, China; Tumor Immunology and Gene Therapy Center, Shanghai Eastern Hepatobiliary Surgery Hospital, Shanghai 200438, China; State Key Laboratory of Genetic Engineering, School of Life Sciences, Zhongshan Hospital, Fudan University, Shanghai 200438, China; Collaborative Innovation Center for Cancer Personalized Medicine, School of Public Health, Nanjing Medical University, Nanjing 211166, China; State Key Laboratory of Genetic Engineering, School of Life Sciences, Zhongshan Hospital, Fudan University, Shanghai 200438, China

**Keywords:** neutrophils, YAP, TAZ, gastric cancer

## Abstract

As an important part of tumor microenvironment, neutrophils are poorly understood due to their spatiotemporal heterogeneity in tumorigenesis. Here we defined, at single-cell resolution, CD44^−^CXCR2^−^ neutrophils as tumor-specific neutrophils (tsNeus) in both mouse and human gastric cancer (GC). We uncovered a Hippo regulon in neutrophils with unique YAP signature genes (e.g., *ICAM1*, *CD14*, *EGR1*) distinct from those identified in epithelial and/or cancer cells. Importantly, knockout of YAP/TAZ in neutrophils impaired their differentiation into CD54^+ ^tsNeus and reduced their antitumor activity, leading to accelerated GC progression. Moreover, the relative amounts of CD54^+ ^tsNeus were found to be negatively associated with GC progression and positively associated with patient survival. Interestingly, GC patients receiving neoadjuvant chemotherapy had increased numbers of CD54^+ ^tsNeus. Furthermore, pharmacologically enhancing YAP activity selectively activated neutrophils to suppress refractory GC, with no significant inflammation-related side effects. Thus, our work characterized tumor-specific neutrophils in GC and revealed an essential role of YAP/TAZ-CD54 axis in tsNeus, opening a new possibility to develop neutrophil-based antitumor therapeutics.

## Introduction

As the most abundant immune cells in human peripheral blood, neutrophils serve as the first line of defense against pathogen infection and tissue injury (Kolaczkowska and Kubes, 2013). Biologists have been able to activate neutrophils more than a century ago. Yet a longstanding dilemma remains: if neutrophils are too suppressed, one dies from infection; if neutrophils are too activated, one dies from autoinflammation. Tumor-associated neutrophils (TANs) are associated—sometimes negatively and sometimes positively—with the progression of various cancers (Shaul and Fridlender, 2019). However, selectively activating neutrophils or certain subset(s) of these cells against tumor cells without damaging normal tissues remain highly challenging. This lack of progress is in large part due to our incomplete understanding of neutrophils.

The specific role of neutrophils in cancer initiation and progression remains a topic of controversy. The antitumor effects of neutrophils include not only cytotoxicity and innate immunity, but also the induction of adaptive immunity (Eruslanov et al., 2014; Finisguerra et al., 2015; Singhal et al., 2016; Mensurado et al., 2018; Ponzetta et al., 2019; Jaillon et al., 2020). For example, neutrophils directly kill tumor cells via reactive oxygen species (ROS) production and H_2_O_2_-mediated cytotoxicity (Granot et al., 2011; Blaisdell et al., 2015; Gershkovitz et al., 2018). The protumor effects of neutrophils range from angiogenesis and extracellular matrix remodeling to metastasis and immune suppression (Nozawa et al., 2006; Shojaei et al., 2008; Spiegel et al., 2016; Albrengues et al., 2018; Szczerba et al., 2019). For example, the DNA component of NETs may act as a chemotactic factor to attract cancer cells for distant metastasis (Cools-Lartigue et al., 2013; Yang et al., 2020). As the third leading cause of cancer-related death worldwide, gastric cancer (GC) is poorly understood and has very limited therapeutic treatment options. In this regard, the specific functions of neutrophils or potential subsets of these cells in GC have not been clearly defined.

The paradoxical phenotypes of neutrophils largely arise from their enormous spatiotemporal heterogeneity resulting from their maturation, activation, half-life, aging status, tissue distribution, and effector function diversity (Ng et al., 2019; Ballesteros et al., 2020; Dinh et al., 2020; Kwok et al., 2020; Németh et al., 2020). Traditionally, tumor-infiltrated neutrophils (tiNeus) are generally referred to as TANs. While most tiNeus appear to feature like tumor-specific neutrophils (tsNeus), some of them could have similar properties to neutrophils found in other tissues such as peripheral blood. To date, the specific subpopulations of tsNeus have not been characterized. In particular, the molecular markers of tsNeus remain unknown, not to mention their antitumor activity and related mechanism. It has been realized that differentiation and maturation of neutrophils are mainly orchestrated by various transcription factors (Evrard et al., 2018a; Xie et al., 2020a). However, the specific transcriptional wiring of tsNeus governing their antitumor activity remains unclear.

The evolutionarily conserved Hippo-YAP pathway has been well established to regulate organ size and tissue homeostasis (Pan, 2010; Zhao et al., 2011; Harvey et al., 2013; Yu et al., 2015). Dysregulation of the Hippo-YAP signaling, in particular, hyperactivation of the transcriptional co-activators YAP and TAZ, has been extensively associated with tumorigenesis, making this pathway a promising therapeutic target. Previously, we discovered VGLL4 as a natural antagonist of YAP and dissected the machinery of PP2A-mediated inactivation of the Hippo kinases MST1/2 in tumor cells; these findings inspired us to develop therapeutic peptides to regress GC (Jiao et al., 2014; Tang et al., 2020). In addition, we and others have revealed important functions of Hippo-YAP pathway in various types of immune cells including macrophage and T cell (Jiao et al., 2015; Geng et al., 2017; Ni et al., 2018). However, it remains elusive whether Hippo-YAP pathway also plays a role in neutrophils, especially in tsNeus during tumorigenesis.

In this work, we identified by single-cell RNA sequencing (scRNA-Seq) analysis a conserved population of tiNeus (CD44^−^CXCR2^−^) as tsNeus and uncovered YAP/TAZ to be essential for the differentiation and antitumor activity of tsNeus in GC. The heterogeneous tsNeus can be divided into four subsets, with CD54^+ ^tsNeus having stronger antitumor activity. Moreover, the relative abundance of CD54^+ ^tsNeus among tiNeus was found to prognosticate better clinical outcomes in GC patients. Also, CD54^+ ^tsNeus dynamically responded to neoadjuvant chemotherapy in GC patients. Mechanistically, a YAP/TAZ-CD54 axis was found to be required for tsNeus development and their tumor-killing ability. Based on these findings, we then developed a therapeutic strategy involving targeting the Hippo-YAP pathway to selectively activate neutrophils against GC without causing excessive damage to normal tissue.

## Results

### Characterization of tsNeus at single-cell resolution in GC

**Figure 1.**
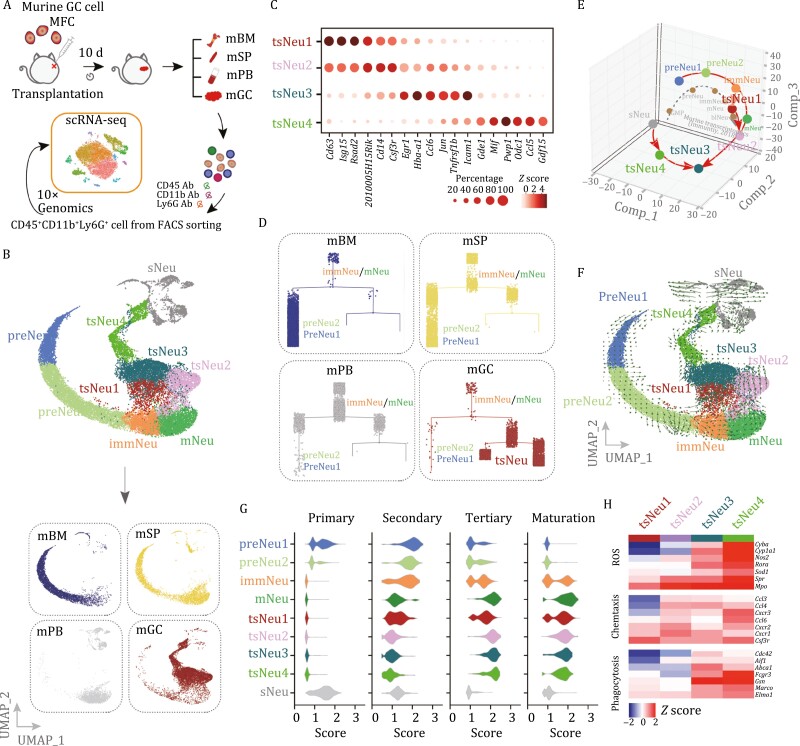
**Identification of tsNeus with distinct transcriptomic signatures in mouse GC.** (A) Schematic illustration of the experimental workflow. Murine CD45^+^CD11b^+^Ly6G^+ ^cells from bone marrow (mBM), spleen (mSP), peripheral blood (mBL), and GC tumors (mGC) were sorted by performing fluorescence-activated cell sorting (FACS) and profiled by carrying out an scRNA-Seq analysis (following the 10× Genomics protocol). Note that subcutaneous mouse GC model was used throughout the manuscript unless otherwise stated. (B) Uniform manifold approximation and projection (UMAP) plots of 22,069 neutrophils from mBM, mSP, mPB, and mGC, and identified using integrated analysis, and colored by cell cluster (upper) and tissue (lower). preNeu1/2, preNeu 1/2; immNeu, immature Neu; mNeu, mature Neu; tsNeu1–4, tumor-specific Neutrophil 1–4; sNeu, silence Neu. (C) Dot plot showing the scaled expression of selected signature genes for the indicated cell clusters (tsNeu1–4), colored by the average expression of each gene in each cluster scaled across all clusters. Dot size represents the percentage of cells in each cluster with more than one read of the corresponding gene. (D) Monocle trajectory prediction for the sequenced neutrophils from the indicated samples. (E) Principal component analysis of gene expression data from neutrophil subsets and available bulk RNA-Seq. (F) Velocity analysis revealing the interrelationship of neutrophil subpopulations. Velocity fields were projected onto the UMAP plots. (G) Violin plots of primary (azurophil), secondary (specific), tertiary (gelatinase score and secretory), and maturation scores for the neutrophil clusters. (H) Heatmap showing the expression of ROS biosynthetic-process-, phagocytosis-, and chemotaxis-related genes in the indicated neutrophil subsets. See also Fig. S1.

To map tissue-specific neutrophils during gastric tumorigenesis, we performed fluorescence-activated cell sorting (FACS) to isolate CD45^+^CD11b^+^Ly6G^+ ^cells from bone marrow (BM), spleen (SP), peripheral blood (PB), and GC tissues of mice (*n* = 3) bearing subcutaneous GC, and performed scRNA-Seq analysis on these cells (Fig. 1A). After excluding dead cells and putative doublets, we obtained 22,069 high-quality cells with an average of 1,189 genes per cell profiled, and 17,458 genes detected in total (Fig. S1A–C and Table S1). Using genome-wide correlations between cluster mean expression and previously defined transcriptional profiles of sorted neutrophil subsets (Evrard et al., 2018b; Kwok et al., 2020; Xie et al., 2020b) and canonical markers (Table S2), we identified nine major cell clusters (Fig. 1B, upper). Note that *Ly6c*^*hi*^*Itgb3*^*hi*^ pre-neutrophils (preNeu1/2) were mainly from bone marrow and spleen, and *Ngp*^*hi*^*Cxcr2*^*lo*^ immature neutrophils (immNeus) and *Cxcr2*^*hi*^*Mmp8*^*hi*^ mature neutrophils (mNeus) were mainly from spleen and peripheral blood (Figs. 1B and S1D). These findings were consistent with the previous studies of neutrophils in steady and bacterial infection states (Evrard et al., 2018b; Kwok et al., 2020; Xie et al., 2020b) and thus verified the robustness of our scRNA-Seq analysis.

In contrast to the overlapping of cell clusters from bone marrow, spleen, and peripheral blood (preNeu1/2, immNeu, mNeu), the transcriptomic map of a major subpopulation of tiNeus appeared to be well separated from that of neutrophils in other tissues; we therefore termed this subpopulation as tsNeus (Fig. 1B, lower). Dimensional reduction with uniform manifold approximation and projection (UMAP) plots further revealed four distinct clusters of tsNeus (Figs. 1B and S1D). Moreover, the tsNeu1/2 clusters were found to be *Cd44*^*lo*^*Icam1*^*lo*^*Gdf15*^*lo*^, whereas the tsNeu3/4 clusters were *Cd44*^*lo*^*Icam1*^*hi*^*Gdf15*^*hi*^ (Figs. 1C and S1D; Table S2). In addition, we also noticed a highly heterogeneous cell cluster consisting of neutrophils from all four examined tissues, and expressing early markers for neutrophil development such as *Elane and Prtn3*; we denoted these neutrophils as silent neutrophils (sNeus) (Figs. 1B and S1D; Table S2).

To resolve the lineage of tsNeus relative to neutrophils in other tissues, we performed a Monocle pseudo-time analysis (Qiu et al., 2017). Consistent with the above UMAP analysis, tsNeus were found to be closely associated with neutrophil subsets in peripheral blood (immNeu and mNeu subsets) but more remotely associated with those in bone marrow and spleen (preNeu1/2) (Fig. 1D). Furthermore, we performed principal component analysis to compare gene expression profiles of all of the above-identified neutrophil subsets. Consistent with previous studies (Evrard et al., 2018b; Xie et al., 2020b), the preNeu1/2, immNeu, and mNeu subsets displayed distinct gene signatures (Fig. 1E). Notably, tsNeu1 and tsNeu2 displayed gene expression profiles similar to those displayed by immNeus and mNeus, respectively (Fig. 1E). Moreover, tsNeu3/4 showed significant transcriptomic differences compared to tsNeu1/2 (Fig. 1E). Interestingly, although sNeus were Ly6G^+^, these neutrophils showed a transcriptional profile similar to that of granulocyte monocytes progenitors (GMPs), which have been well identified to constitute a subset of Ly6G^-^ neutrophils (Fig. 1E).

Neutrophil differentiation has been shown to occur on a tightly organized trajectory, starting from GMP cells in bone marrow and spleen to mNeus in spleen and peripheral blood. A cluster of immNeus followed preNeu expansion and expressed secondary granule genes such as *Ngp* (Fig. 1D), and this cluster migrated from bone marrow to the peripheral blood. Neutrophil differentiation in bone marrow tended to end with mNeus showing high expression of *Mmp8* (Figs. 1D and S1D). An RNA velocity analysis, a method providing information on precursor-progeny cell dynamics, revealed a strong directional flow of preNeu subsets towards immNeus and mNeus (Fig. 1F), indicating that neutrophil maturation (from preNeus to mNeus) follows a single main branch without significant division. We also observed a clear directional flow from immNeus and mNeus enriched in peripheral blood to tsNeus enriched in tumor tissue (Fig. 1F). Additionally, we observed that 7.3% of the sNeus (93 of 1,265) had the potential to transition to tsNeu3/4 (Fig. 1F), suggesting that tsNeu3/4 may originate from both tsNeu1/2 and sNeus.

Neutrophils contain an assembly of granules destined for regulated secretion, and each granule type with distinct constituents has been indicated to form before terminal differentiation (Borregaard et al., 2007). The earliest granules are designated as primary granules, followed in time by secondary and then tertiary granules (Cowland and Borregaard, 2016). We analyzed the expression of various granule genes and found tsNeus displaying a highly coordinated expression of tertiary granules, at a level comparable to that of mNeus (Fig. 1G). We also measured the levels of neutrophil maturation, aging, and apoptosis based on their expression of related genes (Table S3). Overall, the maturation levels of tsNeus were determined to be similar to those of immNeus and mNeus (Fig. 1G). Notably, tsNeu3/4 showed the highest aging and apoptosis scores of all the tsNeus (Figs. 1G and S1E), indicating a terminal-bound identity. tsNeu4 showed the highest expression of key genes involved in ROS production, phagocytosis, and chemotaxis (Fig. 1H).

To investigate the dynamic evolution of tsNeus with tumor progression, we subsequently went on to analyze the change of the tsNeu transcriptome within a time window of 3–10 days. With the same sequencing depth of scRNA-Seq, we observed an increase in both gene number and total unique molecular indexes in neutrophils isolated from mice bearing tumors for 10 days when compared with those from mice bearing tumors for 3 days, indicating elevated transcriptional activity in neutrophils during tumorigenesis (Table S1). Further t-distributed stochastic neighbor embedding (t-SNE) analysis (van der Maaten and Hinton, 2008) also confirmed that the tiNeus were a mixed population of preNeus, immNeus, mNeus, tsNeus, and sNeus (Fig. S1F). Mice bearing GC for 10 days showed relatively lower quantities of tsNeu1/2 but more tsNeu3/4 than did the mice bearing GC for 3 days (Fig. S1F and S1G), indicating a transition of tsNeu1/2 into tsNeu3/4 along with tumor progression.

Taken together, these results defined, at single-cell resolution, tsNeus as a unique population residing only in tumors, that is, not in other tissues—and revealed that tsNeus constitute the endpoint of the trajectory of neutrophil development during the course of tumorigenesis, showing both activated and atavistic features.

### tsNeus are CD44 and CXCR2 double negative

**Figure 2.**
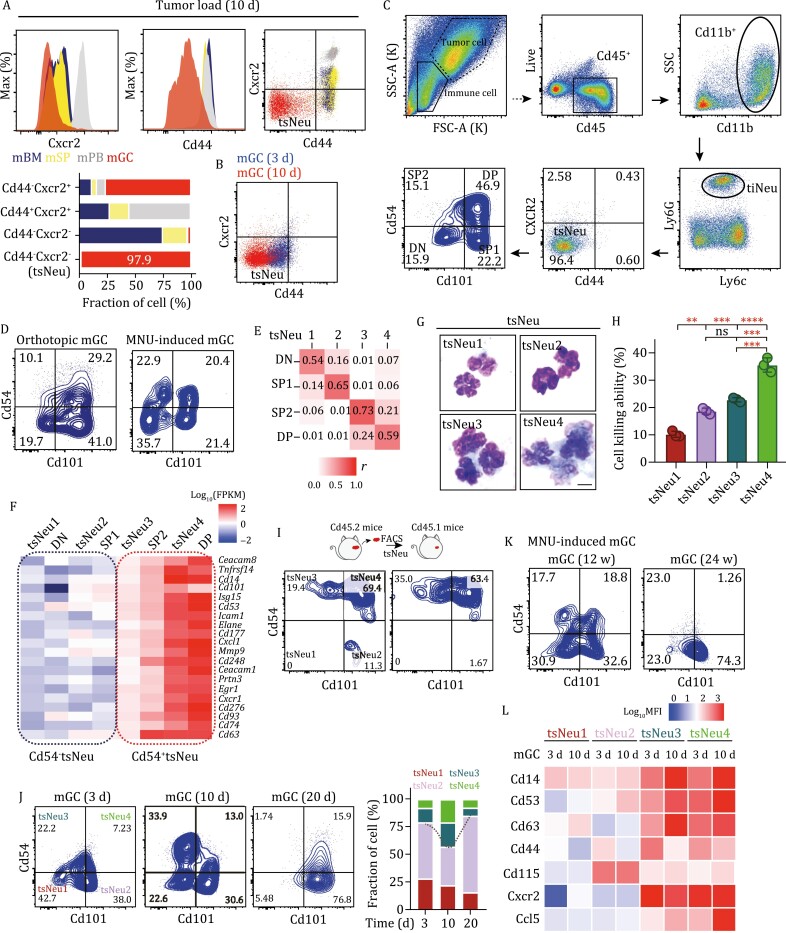
**Characterization of four distinct subpopulations of tsNeus.** (A) Expression levels of CD44 and CXCR2 in Ly6G^+ ^neutrophils from BM (blue), spleen (yellow), peripheral blood (gray), and GC (red). Bar graph shows the relative amounts of CD44^−^CXCR2^−^, CD44^−^CXCR2^+^, CD44^+^CXCR2^−^, and CD44^+^CXCR2^+ ^subsets in four tissues. (B) Expression levels of CD44 and CXCR2 in tiNeus derived from mice after they were subcutaneously implanted with MFC cells for 3 days (blue) or 10 days (red). (C) Representative FACS plots showing the GC neutrophil gating strategy. (D) Flow cytometry plots of tsNeu subpopulations in tumors from the indicated GC models. (E) Correlation matrix generated with Pearson’s correlation coefficients represents similarities of gene expression between subsets (low similarity = white, high similarity = red). (F) Heatmap of the 20 genes most differentially expressed between subsets. (G) Representative images of sorted subpopulations. Scale bar, 10 μm. (H) Killing ability of the indicated tsNeu subsets to kill cocultured gastric tumor cells (MFC). Cytotoxicity was determined from an LDH release assay. (I) Transfer of sorted CD45.2^+ ^tsNeu1/2 or CD45.2^+ ^tsNeu3/4 into CD45.1^+ ^wild-type recipients. Results represent transferred subset after 2 days. (J) tsNeu subpopulations in a subcutaneous mGC model at the indicated number of days (d) after implantation. at the indicated time. (K) tsNeu subpopulations in an MNU-induced GC model at the indicated number of weeks after implantation. (L) Heat map showing the expression levels of the indicated markers in the tsNeus from murine tumor tissues (3 or 10 days). Note: tumor-infiltrated neutrophil, tiNeu; tumor-specific neutrophil, tsNeu; CD54^−^CD101^−^, DN; CD54^−^CD101^+^, SP1; CD54^+^CD101^−^, SP2; CD54^+^CD101^+^, DP. Data in C and D were analyzed using one-way ANOVA, followed by the Tukey’s post hoc test. **P < 0.01; ***P < 0.001; ****P < 0.0001; ns, no significance in comparison with the control group (same below). See also Fig. S2.

We next investigated, at protein levels, the cell identity and state of tsNeus. From the flow cytometric analysis, we observed preNeus (CD11b^+^Gr1^+^CXCR4^+^c-Kit^+^) in tumor-bearing mice showing a gradual upregulation of c-Kit and a subsequent bifurcation branch of c-Kit^− ^neutrophils into CD101^− ^and CD101^+ ^cells, corresponding to the previously characterized immNeus and mNeus, respectively (Fig. S2A) (Evrard et al., 2018b). Given the common origin of Ly6G^+^ly6C^− ^neutrophils, however, these surface markers could not distinguish tsNeus from other types of neutrophils (Fig. S2A and S2B). To overcome this issue, we systematically evaluated the expression levels of various cell surface markers, including neutrophil lineage-specific (c-kit, CD53, CD63, CD101, CXCR2, CXCR4, Ly6G, Ly6C), activation (CD14, CD54), rolling (CD44, CD62L), and trans-endothelial migration (CD11b) markers (Beinert et al., 2000; Metkar et al., 2012; Kolaczkowska and Kubes, 2013). Consistent with our scRNA-Seq analysis indicating an activated but atavistic phenotype, tsNeus showed high expression levels of Ly6G, CD53, CD54 (ICAM1), CD14, CD63, PDL1, and c-Kit, but low expression of Ly6C, CD62L, CD44, and CXCR2 (Figs. 2A, S2B, and S2C). We then performed a mini-screening by gating various cell surface markers. Surprisingly, we observed a clear separation of tsNeus from all other Ly6G^+^ly6C^−^ neutrophils when sorted by CD44 and CXCR2 (Figs. 2A and S2D). Notably, most of the tsNeus (93.2%) were CD44^−^CXCR2^−^, and the majority (97.9%) of the CD44^−^CXCR2^− ^neutrophils were tsNeus (Fig. 2A). Moreover, most neutrophils in the peripheral blood were CD44^+^CXCR2^+^, while neutrophils in the bone marrow and spleen consisted of both CD44^+^CXCR2^− ^and CD44^+^CXCR2^+ ^subsets (Fig. 2A). In contrast, we found that the ratio of the number of CD44^−^CXCR2^− ^tsNeus to the number of tiNeus significantly increased from ~88% in mice bearing GC for 3 days to ~97% in mice bearing GC for 10 days (Fig. 2B). These observations were further confirmed from analyses using an orthotopic mouse GC model (Fig. S2E).

### A combination of CD54 and CD101 marks tsNeu1–4 subsets

After being able to isolate tsNeus, we went on to further dissect their heterogeneity by searching for cell surface markers specific for tsNeu subsets. Given the activated but atavistic features of tsNeus, we chose CD54 (ICAM1) to segregate nonactivated from activated neutrophils, and CD101 to segregate immature from mature neutrophils. As such, we characterized four phenotypically distinct tsNeu subsets (DN: CD54^−^CD10^−^; SP1: CD54^−^CD101^+^; SP2: CD54^+^CD10^−^; DP: CD54^+^CD101^+^) (Fig. 2C), which we speculated might have corresponded to the four subsets defined by our scRNA-Seq analysis (tsNeu1–4). Similarly, we defined four tsNeu subsets based on their expression levels of CD54 and CD101 in the tumor tissues from the orthotopic GC model, as well as from an MNU-induced GC model (Fig. 2D), confirming that CD54 and CD101 can serve as specific cell surface markers to subclassify and isolate tsNeus into four subpopulations.

Next, we performed bulk RNA sequencing of the FACS-isolated tsNeu subsets (DN, SP1, SP2, DP), and compared them with the scRNA-Seq-defined tsNeu subsets (tsNeu1–4). Pearson correlation matrix analysis showed similar gene expression profiles for tsNeu1 and DN, as well as for tsNeu2 and SP1, tsNeu3 and SP2, and tsNeu4 and DP, respectively (Fig. 2E). Moreover, even though DN/SP1 and SP2/DP tsNeus were classified only by CD54 expression, these two subsets showed vast transcriptomic differences with more than 2000 differentially expressed genes including *Cd14* and *Icam1* (*Cd54*) (Fig. 2F). Importantly, morphological analysis with Wright–Giemsa staining revealed a polysegmented shape of the nucleus for tsNeu1 (immature-like shape) and tsNeu2 (mature-like shape), with progressively increased cell surface areas for tsNeu3 and tsNeu4 (Figs. 2G and S2F). We also performed an LDH release assay to determine tsNeu-induced cytotoxicity toward tumor cells. As expected, treatment of MFC cells, from a murine GC cell line, with tsNeu1 for 48 h induced ~10% increase in the death of the MFC cells (Fig. 2H). Notably, CD54^+ ^tsNeus (tsNeu3/4) showed a stronger antitumor activity than did CD54^− ^tsNeus (tsNeu1/2) (Fig. 2H), findings consistent with the results of our scRNA-Seq analysis.

### Differentiation and dynamics of tsNeu subsets during GC progression

To determine the differentiation route of tsNeu subsets during tumorigenesis, we isolated CD54^− ^and CD54^+ ^tsNeus from CD45.2 mice bearing subcutaneous GC, and then adoptively transferred them into the tumors borne by CD45.1 mice. After 48 h, most of the CD54^− ^tsNeus became CD54^+ ^tsNeus, whereas all of the CD54^+ ^tsNeus remained CD54^+^—findings consistent with the scRNA-Seq results indicating the ability of tsNeu1/2 to give rise to tsNeu3/4 but the lack of a transformation in the reverse direction (Fig. 2I). Moreover, annexin V/PI staining showed a higher rate of apoptosis for CD54^+ ^tsNeus than CD54^− ^tsNeus, also suggesting a more terminal status for tsNeu3/4 relative to tsNeu1/2 (Fig. S2G).

In keeping with the scRNA-Seq results (Fig. S1F and S1G), we subsequently examined tiNeus (CD11b^+^Ly6G^+^) in mice bearing subcutaneous gastric tumors for 3, 10, and 20 days, and found that the proportion of tiNeus decreased rapidly with tumor progression, from 53.1% on day 3 to 6.02% on day 20 (Fig. S2H). These observations indicated a correspondence of the time points of 10 and 20 days to early and advanced stages of tumorigenesis, respectively. Moreover, CD54^+ ^tsNeu (tsNeu3/4) levels were greater in mice bearing GC for 10 days than in those bearing GC for 3 days, and in those bearing GC for 20 days (Fig. 2J). In an MNU-induced mouse GC model, we similarly observed a sharp decrease in CD54^+ ^tsNeu levels from the time point of 12 weeks (early stage) to the time point of 24 weeks (late stage) (Fig. 2K). Meanwhile, the expression levels of CCL5, CD14, CD53, and CD63 in CD54^+ ^tsNeus were increased from the time point of 3 days to 10 days in mice bearing subcutaneous GC (Fig. 2L).

In addition, we observed no significant change for CD115^+^Ly6G^+ ^cells, a previously reported population of MDSCs (Huang et al., 2006; Youn et al., 2012) (Fig. S2I). Also, we used early neutrophil lineage markers CD34 and c-Kit to isolate sNeus, and found that the proportion of sNeus was significantly reduced from ~2.0% in mice bearing GC for 3 days to 0.27% in mice bearing GC for 10 days (Fig. S2J); these findings are consistent with our scRNA-Seq analysis.

### CD54^+ ^tsNeu abundance is negatively correlated with human GC progression and positively correlated with patient survival

**Figure 3.**
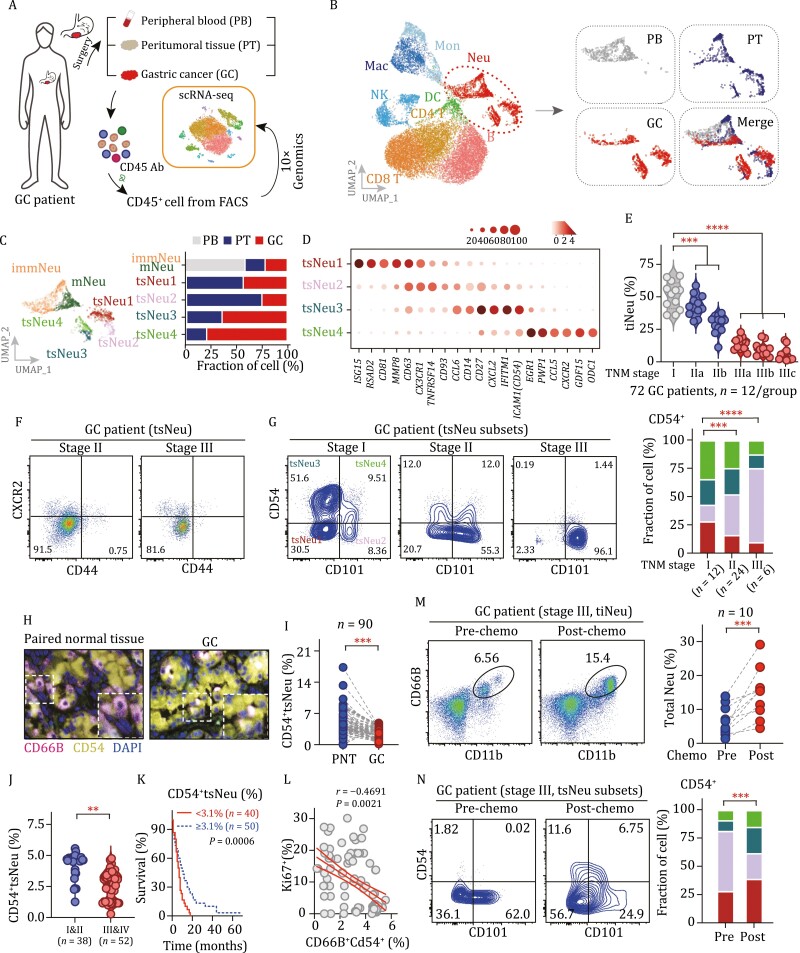
**tsNeus are present in humans and associated with GC progression.** (A) Experimental workflow. FACS-isolated CD45^+ ^cells from the peripheral blood (PB), peritumoral tissue (PT) and gastric cancer tissue from GC patients and profiled by carrying out an scRNA-Seq analysis (10× Genomics). (B) UMAP plots of total CD45^+ ^immune cells colored by cluster (left) and tissue (right). (C) UMAP plot showing neutrophil subsets. Bar graph showing the distributions of the indicated tsNeu subsets in the three indicated tissues. (D) Dot plot showing the expression levels of selected signature genes for the indicated tsNeu clusters. (E) Violin plots showing the relative amounts of tiNeus (CD11b^+^CD66b^+^) in human GC tissues for various tumor progression stages. (F) Relative amount of tsNeus in the tumor tissues from GC patients with indicated tumor stages. (G) Expression plots of CD54 and CD101 denoting the various tsNeu subpopulations. Representative flow cytometric analysis plots are shown. A bar graph showing the distributions of the tsNeu subpopulations in the GC tissues. (H) Representative images showing the staining of CD66b and CD54 (ICAM1) on tissue microarrays derived from 90 GC patients. Bar, 100 μm. (I) Quantification of total CD54^+ ^tsNeu (CD66b^+^CD54^+^) from GC immunohistochemistry (IHC) microarrays. Staining (%) was calculated from IHC integrated optical density (IOD). Paired *t-*test. (J) Quantification of CD54^+ ^tsNeus (%) in GC patients with indicated tumor stages. Unpaired *t-*test. (K) Kaplan–Meier survival analysis of patients with high or low fractions of CD54^+ ^tsNeus (%). Log-rank test. (L) Scatter plot of the relative amounts of CD54^+ ^tsNeus (%) versus Ki67^+ ^tsNeus (%) in GC patients. Spearman’s correlation analysis. (M) Quantification of tiNeus in GC patient tumor tissues before or after neoadjuvant chemotherapy. Biopsy samples were endoscopically obtained from 10 GC patients (stages II and III) pre and post neoadjuvant chemotherapy. (N) tsNeu subpopulations in tumors from GC patient before or after the patients received neoadjuvant chemotherapy. See also Fig. S3.

To determine possible human equivalents of tsNeus, we performed an scRNA-Seq analysis of CD45^+ ^cells isolated from clinical samples of two GC patients (Fig. 3A). The GC tissue, PT, and PB were from four patients who had not yet received cancer treatment. A total of 20,985 cell transcriptomes passed quality control, with 3,357 of them for neutrophils and the rest for other types of cells including monocytes (1,176), macrophages (1,859), T cells (6,266), NK cells (1,506), B cells (3,969), and dendritic cells (894) (Fig. 3B). Ligand–receptor interaction analysis (Ren et al., 2020) revealed a high affinity of neutrophils for monocytes in the tumor microenvironment (Fig. S3A). Subsequent spectral clustering resolved neutrophils into six subsets, namely immNeu, mNeu, and tsNeu1–4 subsets (Fig. 3C). Notably, the relative proportions of these neutrophil subsets were found to vary considerably in different tissues: immNeu and mNeu subsets were enriched ~3-fold in PB in comparison to PT and GC; tsNeu1/2 were increased >10-fold in PT and GC in comparison to PB; tsNeu3/4 were exclusively present in GC (Fig. 3C).

To further clarify the relationship between the neutrophil subsets in mice and their equivalents in humans, we compared their transcriptional signatures across species. Regardless of species, tsNeu1/2 expressed high amounts of neutrophil lineage markers (*CD63* and *CD81*) and type I interferon genes (ISG15 and RSAD2), whereas tsNeu3/4 tended to express activation markers (*ICAM1* and *CCL5*) (Figs. 1C and 3D). Consistently, both mouse and human tsNeus were found to display highly coordinated expression of tertiary granules, with tsNeu3/4 showing higher scores of maturation, aging, and apoptosis than tsNeu1/2 (Fig. S3B–D). Moreover, trajectory analysis showed a common ordering of neutrophil subsets from tsNeu1 via tsNeu2 to tsNeu3 and tsNeu4 in either mice or humans (Fig. S3E). These observations indicated a transcriptomic similarity between the mouse and human equivalents of tsNeu subsets.

Next, we performed flow cytometric analysis to examine, at the protein level, the similarity between mouse and human tsNeus. Similar to the observations in mouse GC models, the relative amount of human tiNeus (CD11b^+^CD66b^+^) was also found to rapidly decrease with tumor progression (from 51.2% at stage I to 5.91% at stage IIIc) (Fig. 3E). Most tiNeus were tsNeus (CD44^−^CXCR2^−^) in human GC samples (Fig. 3F). Like for mouse tsNeus, four subsets of human tsNeus were also identified (tsNeu1: CD101^−^CD5^−^, tsNeu2: CD101^+^CD54^−^, tsNeu3: CD10^−^CD54^+^, tsNeu4: CD101^+^CD54^+^) (Figs. 3G and S3F). Also, with tumor progression from stage I to stage III, the fractions of tsNeu3/4 (CD54^+ ^tsNeus) decreased while that of tsNeu2 increased (Figs. 3G and S3F). These findings further verified our notion that tsNeus are conserved from mice to human.

To further assess the pathological significance of tsNeu3/4 (CD54^+ ^tsNeus), we processed data from 72 GC patients by extracting nine available clinical factors in three categories, that is, the clinical background (age, gender), immunohistochemistry data (Lauren classification, differentiation, lymphatic invasion), and the cancer stage information (tumor size, lymph node metastasis, distant metastasis, tumor stage). Considering the important role of *Helicobacter pylori* infection in gastric tumorigenesis, we also included *H*. *pylori* infectious status for patient analysis. According to the results, the abundance of CD54^+ ^tsNeus among tiNeus was negatively correlated not only with lymphatic invasion and lymph node metastasis, but also with tumor size and tumor stage (Table 1). These observations were also confirmed by the results of staining of CD54^+ ^tsNeus from tissue microarrays containing 90 GC specimens having a long-term clinical follow-up record (Fig. 3H–J and Table 2). In the subsequent Kaplan–Meier survival analysis, staining of CD54^+ ^tsNeus was positively related to 5-year survival rate of GC patients (Fig. 3K). Importantly, the staining of CD54^+ ^tsNeus was negatively correlated with the staining of Ki67^+ ^cells (Fig. 3L).

**Table 1.** Pathological association of CD54^+ ^tsNeus with human GC (FACS).

Groups	CD54^+ ^tsNeu/tiNeu (%)		*n*	*P*-value (*Fisher*’*s test*)
	<25%	≥25%		
Age (years)				
<60	16	23	39	0.2369
≥60	19	14	33	
Gender				
Male	16	26	42	0.0551
Female	19	11	30	
*Helicobacter pylori*				
Positive	18	27	45	0.0088^*^
Negative	17	10	27	
Lauren				
Intestinal	20	28	48	0.1341
Nonintestinal	15	9	24	
Differentiation				
Low	20	24	44	0.6295
Moderate or high	15	13	28	
Lymphatic invasion				
Ly0–1	13	27	40	0.0041^*^
Ly2–3	22	10	32	
Tumor size				
pT1 + pT2 (*≤*5 cm)	13	25	38	0.0176^*^
pT3 (>5 cm) + pT4	22	12	34	
Lymph node metastasis				
N0 + N1	19	32	51	0.0040^*^
N2 + N3	16	5	21	
Distant metastasis				
M0	35	37	72	1.0000
M1	0	0	0	
Tumor stage				
Stage I + Stage II	12	24	36	0.0050^*^
Stage III + Stage IV	23	13	36	
Total	35	37	72	

Note: Fisher’s exact test was used to test the association between two categorical variables.

^*^Statistically significant, *P* < 0.05.

**Table 2.** Association of CD54^+ ^tsNeu with GC progression (IHC microarray).

Groups	CD54^+ ^tsNeu/tiNeu (%)		*n*	*P*-value (Fisher’s test)
	<25%	≥25%		
Age (years)				
<60	15	10	25	0.0967
≥60	25	40	65	
Gender				
Male	29	31	60	0.3699
Female	11	19	30	
Lymphatic invasion				
Ly0–1	7	20	27	0.0229^*^
Ly2–3	33	30	63	
Tumor size				
pT1 + pT2 (*≤*5 cm)	1	12	13	0.0051^*^
pT3(>5 cm)	39	38	77	
Lymph node metastasis				
N0 + N1	11	25	36	0.0335^*^
N2 + N3	29	25	54	
Distant metastasis				
M0	36	49	85	0.1672
M1	4	1	5	
Tumor stage				
Stage I + Stage II	10	28	38	0.0050^*^
Stage III + Stage IV	30	22	52	
Total	40	50	90	

Note: Fisher’s exact test was used to test the association between two categorical variables.

^*^Statistically significant, *P* < 0.05.

GC is often treated with neoadjuvant chemotherapy. Therefore, we examined whether such clinical treatment may induce significant changes in tsNeus. To this end, we intraperitoneally injected tumor-bearing mice (*n* = 10) with 5-fluorouracil (5-FU, 20 mg/kg) every other day. We observed significantly increased proportions of both total neutrophils and CD54^+ ^tsNeus in tumor tissues after injecting the 5-FU three times (Fig. S3G and Fig. S3H). In addition, we collected samples from 10 GC patients before and after they received neoadjuvant chemotherapy. Similarly, the proportions of both tiNeus and CD54^+ ^tsNeus were significantly increased in GC patients receiving neoadjuvant chemotherapy (Fig. 3M and 3N).

### CD54 is one of the YAP signature genes in neutrophils

**Figure 4.**
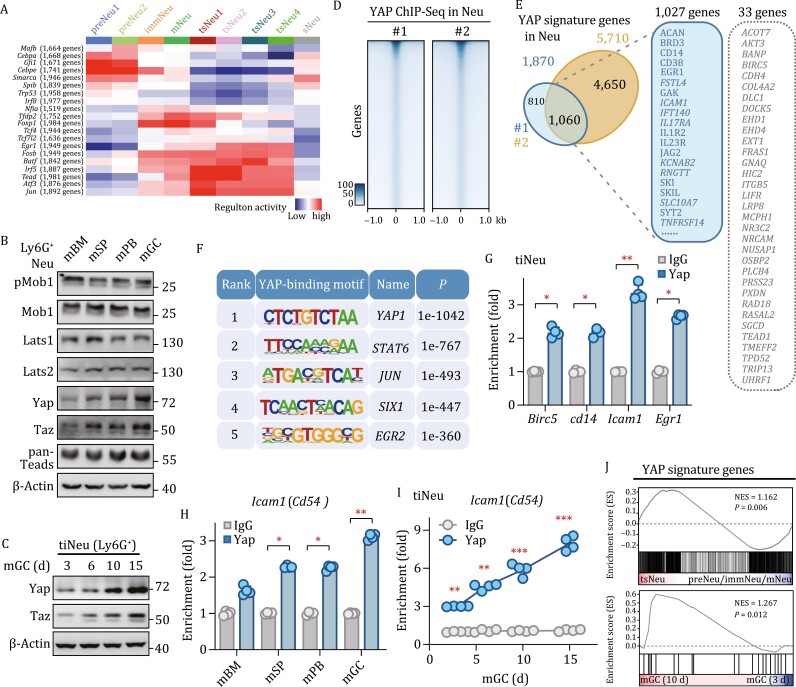
Identification of the Hippo regulon with unique signature genes in tsNeus. (A) Heatmap of regulon activities derived from different neutrophil subsets. Only regulons with *t-*values >100 are shown. (B) Immunoblotting showing the expression levels of pYAP^S127^, pTAZ^S89^, YAP, TAZ, pMOB1, MOB1, LATS1/2, and TEADs in Ly6G^+ ^neutrophils from the indicated tissues. (C) Immunoblotting showing the expression levels of YAP and TAZ in tumor-infiltrated neutrophils (tiNeus) from mice bearing tumors for the indicated periods of time. (D) Heat map representing YAP-binding sites located on promoters. ChIP assays were performed with anti-YAP antibodies from Abcam (#1, ab39361) and Santa Cruz (#2, sc-A2319) in human CD66^+ ^neutrophils, followed by ChIP-Seq. (E) Overlap of signature genes identified by performing ChIP-Seq with YAP #1 and YAP #2 antibodies. Cyan box: 1,027 newly identified YAP signature genes in neutrophils. Gray box: 33 previously identified direct targets of YAP in epithelial and/or cancer cell lines. (F) Motif enrichment analysis of YAP-binding motifs. (G) ChIP assay validating the binding of YAP to the promoters of the indicated genes in tiNeus. (H) ChIP-QPCR showing YAP bound to the *Icam1* promoter in the indicated tissues from tumor-bearing mice. (I) ChIP-QPCR showing YAP bound to the *Icam1* promoter in tiNeu from mice bearing tumors for the indicated periods of time. (J) GSEA analysis showing significant enrichment of YAP signatures in tsNeu subsets. See also Fig. S4.

To gain insights into the molecular machinery governing tsNeus, we performed a single-cell regulatory network inference and clustering (SCENIC) analysis (Aibar et al., 2017) to compare the regulon activities of tsNeus with those of other neutrophil subsets (Fig. 4A). tsNeus highly expressed *Egr1*, *Fosb*, *Batf*, *Irf5*, *Atf3*, and *Jun* regulons previously shown to be associated with neutrophil activation (Xie et al., 2020b) (Fig. 4A). We noticed that tsNeus also displayed high activity of *Teads*, transcription factors of the Hippo-YAP signaling pathway (Pan, 2010; Zhao et al., 2011; Harvey et al., 2013; Yu et al., 2015) (Fig. 4A). The expression pattern of *Teads* was similar to those of *Atf3* and *Jun* (Fig. S4A). Consistent with these results, neutrophils in the tested tumor tissue showed the highest protein levels of Yap/Taz when compared with neutrophils from other tissues including bone marrow, spleen, and peripheral blood (Fig. 4B). Moreover, the expression levels of Yap/Taz in tiNeus seemed to progressively increase with time following tumor implantation (Fig. 4C). Interestingly, the tsNeu1/2 subsets appeared to express higher levels of Yap/Taz than did tsNeu3/4 (Fig. S4B).

In contrast to their well-characterized targets in epithelial and cancer cells (Zhao et al., 2008; Zhang et al., 2009; Zanconato et al., 2015), signature genes of Yap/Taz have not been identified in neutrophils. Therefore, we performed chromatin immunoprecipitation (ChIP) with YAP antibodies (#1: Abcam; #2: Santa Cruz) in CD66b^+ ^cells isolated from the peripheral blood of a GC patient, followed by ChIP-Seq (Fig. 4C). Analysis of the distribution of YAP-binding sites relative to genes annotated in the human genome revealed that only a minute fraction of peaks mapped close to (within 1 kb of) transcription start sites (TSSs) (Fig. 4D), whereas most peaks were located farther than 10 kb from the closest TSS (Fig. S4D). Totals of 1,870 and 5,710 peaks were identified by #1 and #2 anti-YAP antibodies, respectively; and 1,060 peaks were identified by both (Fig. 4E). In addition to 33 direct YAP targets (such as *BIRC5*, *CDH4*, *TEAD1*) previously established in epithelial and cancer cells, 1,027 genes were newly identified to be YAP signatures in neutrophils (Table S4). Some of these new signature genes, for example, *ICAM1* (CD54), *CD14*, *EGR1*, have been previously shown to be essential for neutrophil activation. Further motif analysis showed not only an enrichment of the canonical Yap-binding motif in the ChIPed sequences, but also of STAT6-, JUN-, SIX1-, and EGR2-binding motifs, indicating a coregulatory machinery for these new signature genes in neutrophils (Fig. 4F).

To corroborate our ChIP-Seq analyses, we performed the ChIP assay to examine the occupancies of Yap on *Icam1* (Cd54), *Cd14*, and *Egr1* loci, and found that Yap exhibited a strong ability to bind to these gene loci (Fig. 4G). Consistent with the protein levels of Yap in neutrophils from different tissues, enrichment of Yap on *Icam1* loci was the highest in neutrophils from the tumor tissue when compared with those from other tissues including bone marrow, spleen, and peripheral blood (Fig. 4H). Moreover, Yap enrichment on *Icam1* loci progressively increased with time following tumor implantation (Fig. 4I). Subsequent gene set enrichment analysis (GSEA) revealed a significant positive enrichment of Yap signature genes in tsNeus relative to other neutrophil populations including preNeus, immNeus, and mNeus (Fig. 4J). Also, Yap signature genes were enriched at 10 days relative to those at 3 days (Fig. 4J). Note that many of these positively enriched genes, such as *ICAM1* (CD54) and *CD14*, have been implicated in the specification, activation, and function of neutrophils (Table S5).

### Yap/Taz-Cd54 axis is essential for tsNeu differentiation and antitumor activity

**Figure 5.**
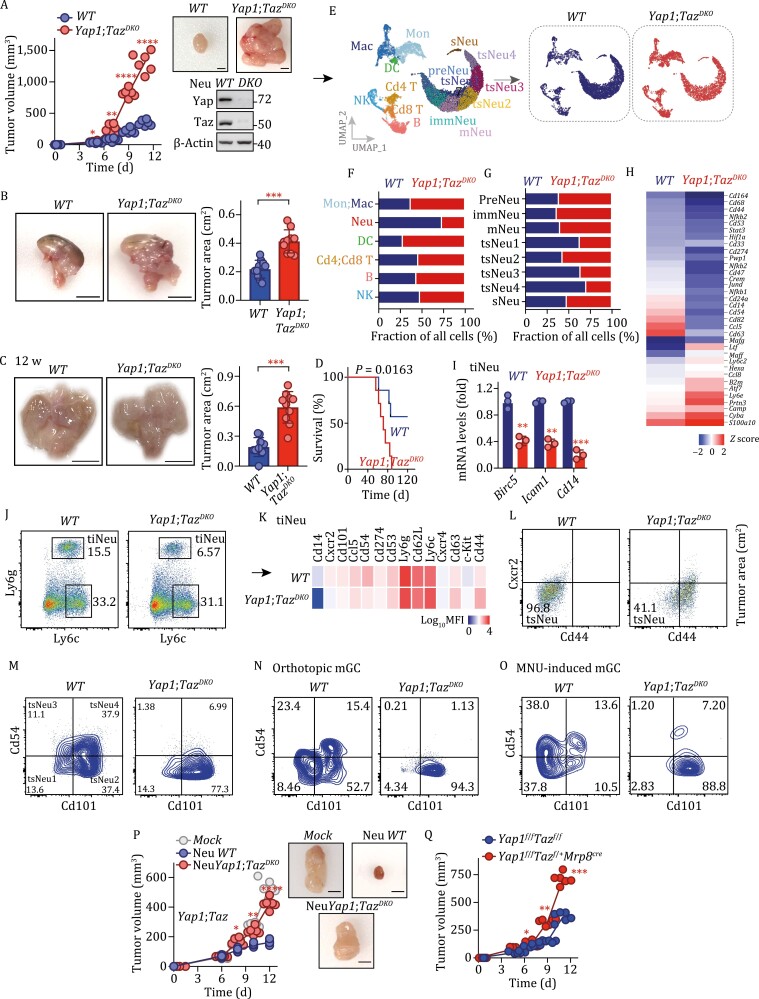
Deficiency of YAP/TAZ decreases the number and antitumor activity of tsNeus. (A) Tumor formation of MFC cells in *Yap1*^*flox*/*flox*^*Taz*^*flox*/*flox*^*LyzM*^*cre*/*cre*^ mice and control mice. Scale bars, 5 mm. (B) Orthotopic tumor formation of MFC cells in *Yap1*^*flox*/*flox*^*Taz*^*flox*/*flox*^*LyzM*^*cre*/*cre*^ mice (*Yap1*;*Taz*^*DKO*^) and control mice (*WT*). (C) MNU-induced tumor formation in *Yap1*^*flox*/*flox*^*Taz*^*flox*/*flox*^*LyzM*^*cre*/*cre*^ mice (*Yap1*;*Taz*^*DKO*^). and control mice (*WT*). (D) Kaplan–Meier survival curves for MNU-treated *Yap1*^*flox*/*flox*^*Taz*^*flox*/*flox*^*LyzM*^*cre*/*cre*^ (*Yap1*;*Taz*^*DKO*^) and control (*WT*) mice. Survival analysis was performed using the log-rank test. (E) UMAP plots of 10,377 CD45^+ ^cells from murine tumors, and workflow of the scRNA-Seq analysis done on murine CD45^+ ^cells from the tumor samples. (F) Bar graph showing the relative amounts of the indicated subsets. (G) Bar graph showing the relative amounts of tiNeu clusters. (H) Heatmap showing the transcription levels of Neu-related genes in tiNeu clusters from *Yap1*^*flox*/*flox*^*Taz*^*flox*/*flox*^*LyzM*^*cre*/*cre*^ mice and control mice. (I) QPCR showing the transcription levels of the indicated genes in tiNeus from *Yap1*^*flox*/*flox*^*Taz*^*flox*/*flox*^*LyzM*^*cre*/*cre*^ mice and control mice. (J) Flow cytometric analysis of tiNeus (CD11b^+^Ly6G^+^Ly6C^lo^) in tumor samples from the *Yap1*^*flox*/*flox*^*Taz*^*flox*/*flox*^*LyzM*^*cre*/*cre*^ mice after they were subcutaneously implanted with MFC cells for 10 days. (K) Heatmap showing the expression levels of the indicated markers in tiNeus from murine tumor tissues. (L) Flow cytometric plots of tsNeus and their subsets from murine tumor samples. (M–O) Flow cytometric plots of tsNeu subsets from murine tumor samples in the indicated GC models. (P) Adoptive transfer assay of neutrophils to MFC-derived tumors. Scale bars, 5 mm. (Q) In vivo tumor formation in *Yap1*^*f*/*f*^*Taz*^*f*/+^*Mrp8*^*cre*/+^ mice and *Yap1*^*f*/+^*Taz*^*f*/+^ mice (*n* = 6). See also Fig. S5.

To assess the importance of the Hippo regulon in neutrophils, we cross-bred *LyzM*^*Cre*^ mice with *Yap1*^*flox*/*flox*^ and *Taz*^*flox*/*flox*^ mice (termed as *Yap1*;*Taz*^*DKO*^). Without any tumor burden, we observed no significant difference in either development or total number of neutrophils between the *Yap1*;*Taz*^*DKO*^ mice and wild-type control littermates (Fig. S5A). However, after subcutaneously inoculating the mice with the same amount of MFC cells, we observed much larger tumors in *Yap1*;*Taz*^*DKO*^ mice than in control mice (Fig. 5A). Similar results were obtained in the orthotopic xenograft and MNU-induced GC mouse models (Fig. 5B and 5C). Of particular interest, we found that relative to the wild-type mice, the *Yap1*;*Taz*^*DKO*^ mice were much more sensitive to the lethal effect of MNU treatment (Fig. 5D), indicating an essential role for Yap/Taz in neutrophil-mediated protection against carcinogen-induced damage and tumorigenesis.

We then inoculated the *Yap1*;*Taz*^*DKO*^ mice (*n* = 2) with MFC cells for 10 days, followed by isolation of a total of 10,858 CD45^+ ^cells for scRNA-Seq (Fig. 5E). After a rigorous quality control, 10,377 single-cell transcriptomes were retained, with 5,847 of them for neutrophils and the rest for other cells including monocytes and macrophages (2,088), T cells (1,772), NK cells (381), B cells (177), and dendritic cells (112) (Fig. 5F). Neutrophils made up a much lower percentage of total immune cells in the *Yap1*;*Taz*^*DKO*^ mice than in the control mice (Fig. 5E and 5F). Importantly, Yap/Taz deficiency led to decreased populations of tsNeus1/3/4 cells but increased populations of tsNeu2, preNeu, immNeu, and mNeu cells (Fig. 5G). Overall, deletion of *Yap1/Taz* resulted in downregulation of activation-related genes such as *CD14*, *CD54* (*ICAM1*), *JunD*, and *Nfkb1*, but upregulation of early lineage-related genes such as *Hexa*, *Ly6c2*, and *Prnt3* (Figs. 5H and S5B). Consistent with these results, a QPCR assay showed significantly decreased mRNA levels of signature genes *Birc5*, *Icam1*, and *Cd14* in Yap/Taz-deficient neutrophils than in wild-type neutrophils (Fig. 5I).

Next, we used FACS to further verify the regulatory effects of Yap/Taz on neutrophils in mice bearing subcutaneous GC. Consistent with the above scRNA-Seq analyses, the *Yap1*;*Taz*^*DKO*^ mice showed a significantly lower relative amount of tiNeus (Cd11b^+^Ly6G^+^Ly6C^lo^) (Figs. 5J and S5C), but a significantly higher relative amount of tumor-infiltrated preNeus (c-kit^+^CXCR4^+^) (Fig. S5D) than did the wild-type mice. Meanwhile, Yap/Taz deficiency did not alter the quantities of GMPs, preNeus, immNeus, and mNeus relative to each other in the bone marrow of tumor-bearing mice (Fig. S5E). Note that Yap/Taz deficiency reduced the expression levels of activation markers such as CD54 and CD14, but enhanced the levels of early neutrophil lineage markers such as CD63 and c-Kit (Fig. 5K). Moreover, the ratio of the population of tsNeus to the population of tiNeus decreased from ~97% in control mice to ~41% in *Yap1*;*Taz*^*DKO*^ mice (Fig. 5L). Similar to the scRNA-Seq results, the population of CD54^+ ^tsNeus was much lower in the *Yap1*;*Taz*^*DKO*^ mice than in the wild-type control mice (Fig. 5M); these findings also confirmed by analyses in both orthotopic xenograft and MNU-induced GC mouse models (Fig. 5N and 5O).

To rule out the possibility that such results were caused by the nonspecific effects of *LyzM*^*Cre*^, we adoptively transferred wild-type or Yap1/Taz-deficient neutrophils into wild-type tumor-bearing mice (Fig. S5F). After carrying out the adoptive transfer three times, tumors receiving wild-type but not Yap1/Taz-deficient neutrophils significantly shrank in volume (Fig. 5P). Furthermore, we also used granulocyte lineage-specific *Mrp8*^*Cre*^ mice to more specifically deplete Yap/Taz in neutrophils (Elliott et al., 2011; Finisguerra et al., 2015). With subcutaneous inoculation of the same amount of MFC cells, tumors grew much larger in the *Yap1*^*f*/*f*^*Taz*^*f*/+^*Mrp8*^*cre*/+^ mice than in control mice (Figs. 5Q and S5G).

### Targeting Hippo-YAP pathway selectively activates neutrophils against mouse GC

**Figure 6.**
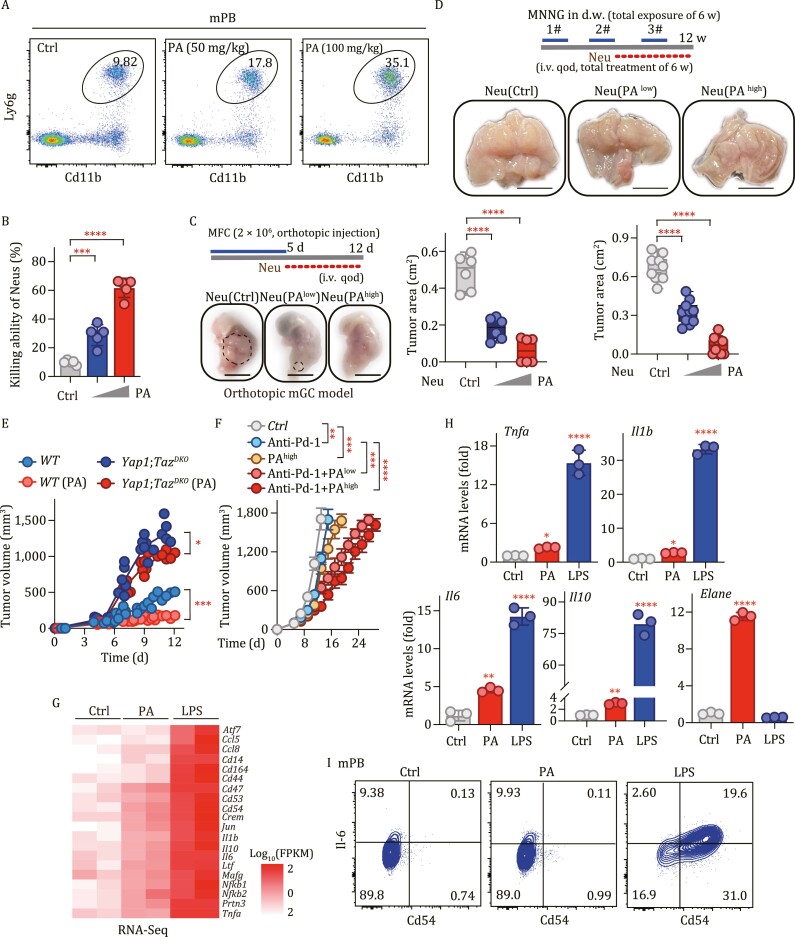
Targeting Hippo selectively activates neutrophils to regress mouse GC. (A) Number of Ly6G^+ ^neutrophils in the peripheral blood (PB) of mice treated with PA for 3 days. (B) *In vitro* tumor-killing ability of Ly6G^+ ^neutrophils from mice treated with PA, with the bar graph showing, as percentages, the neutrophil tumor-killing activities (*n* = 5). (C) Orthotopic tumor formation of MFC cells in mice receiving adoptively transferred PA-treated neutrophils’ tumor surface areas are shown in the right panel. Bar, 1 cm. (D) MNNG-induced tumor formation in mice after transferring with PA-treated neutrophils. Photographs of dissected stomachs (opened along the greater curvature) from mice. The tumor surface area is shown in the right panel. Bar: 1 cm. (E) *In vivo* tumor formations in PA-treated wild-type and *Yap1*;*Taz*^*DKO*^ mice (*n* = 6). (F) Tumor volume results for a combined therapy (PA and anti-PD-1) for treating melanoma (*n* = 10). PA, four times; anti-PD-1, four times. (G) Heat map for RNA-Seq-derived mRNA levels of indicated genes in mouse neutrophils treated with PA or LPS. (H) QPCR-derived mRNA levels of the indicated genes in mouse neutrophils treated with PA or LPS. (I) Protein expression of IL-6 and CD54 in mouse neutrophils treated with PA or LPS. See also Fig. S6.

Given the role of the Hippo-YAP signaling in neutrophils, we reasoned that pharmacologic activation of Yap/Taz may redirect neutrophils against cancer. To test this hypothesis, we treated mice with phosphatidic acid (PA), a Hippo-YAP pathway regulator that activates Yap/Taz, via intraperitoneal injections (50 and 100 mg/kg). After 3 consecutive days of PA injections, we observed an increase in the total number of neutrophils in the blood (Fig. 6A). Importantly, neutrophils isolated from the blood of these PA-treated mice showed significantly increased tumor-killing ability (Fig. 6B). We then examined *in vivo* the PA-mediated activation of neutrophils against tumor growth. First, we tested PA treatment in a nude mice xenograft model and observed an inhibitory effect of this treatment on tumor growth (Fig. S6A). Moreover, with this treatment, we observed increased relative amounts of both total tsNeus (Fig. S6B) and CD54^+ ^tsNeus (Fig. S6C). In a subsequent adoptive transfer study, tumor-bearing mice receiving neutrophils from PA-treated mice had much smaller tumors than did the control group in both orthotopic xenograft and MNU-induced GC mouse models (Fig. 6C and 6D).

To verify whether the PA-induced increase of neutrophil tumor-killing ability is dependent on the Hippo regulon, we treated wild-type and *Yap1*;*Taz*^*DKO*^ mice with PA (100 mg/kg) for 3 consecutive days. PA treatment induced an approximately 2-fold increase in the population of neutrophils in the peripheral blood of wild-type but not *Yap1*;*Taz*^*DKO*^ mice (Fig. S6D). Moreover, PA treatment markedly increased the tumor-killing ability of wild-type but not *Yap1*;*Taz*-deficient neutrophils (Fig. S6E). Notably, we only observed a marginal effect of PA treatment on tumor volume of *Yap1*;*Taz*^*DKO*^ mice (Fig. 6E), again indicating a dependence of the PA-induced tumor-killing effect on the Hippo regulon. Consistent with these results, PA treatment significantly enhanced the transcription of Yap signature genes *Icam1* and *Birc5* in tsNeus from wild-type mice; whereas the expression levels of these signature genes remained at almost basal levels in tsNeus from *Yap1*;*Taz*^*DKO*^ mice (Fig. S6F).

In addition, we observed no significant difference in CD3/CD28-stimulated activation of CD8^+ ^T cells between PA-treated mice and -untreated control mice (Fig. S6G). We then further tested the effect of PA in combination with anti-PD-1 antibody in a mouse xenograft tumor model, in which MFC cells were subcutaneously injected 5 days before the treatment. As expected, the combined therapy showed a better efficacy than monotherapies in inhibiting tumor progression (Fig. 6F).

We next assessed the specificity, selectivity, and potential side effects of the Hippo-YAP-targeting neutrophil immunotherapy. To this end, we performed an RNA-Seq analysis of mouse neutrophils treated with PA or LPS (Fig. S6H). Our results showed that PA-induced gene expression in a pattern distinct from that of LPS signaling (Fig. 6G). We also performed a QPCR analysis using these neutrophils to compare the pattern of transcription of antimicrobial and/or inflammatory cytokines upon treatment with PA versus that upon treatment with LPS. As shown in Fig. 6H, LPS treatment robustly enhanced the mRNA expression levels of *Tnfa*, *Il1b*, *Il6*, and *Il10*, whereas PA treatment only slightly increased the transcription of these cytokines. To our surprise, however, treatment with PA but not LPS substantially increased the expression of Elane, an enzyme that was recently demonstrated to selectively kill cancer cells and to attenuate tumorigenesis (Cui et al., 2021) (Fig. 6H). Consistent with these observations, the protein level of IL-6 was also markedly lower in PA-treated neutrophils than in LPS-treated neutrophils (Fig. 6I). Note that PA did not stimulate the expression of CD54 neutrophils in the absence of tumor cells, suggesting that unlike LPS signaling, PA-induced activation of neutrophils requires their exposure to tumor cells (Fig. 6I).

### The Hippo-YAP-targeting neutrophil therapy regresses refractory human GC

**Figure 7.**
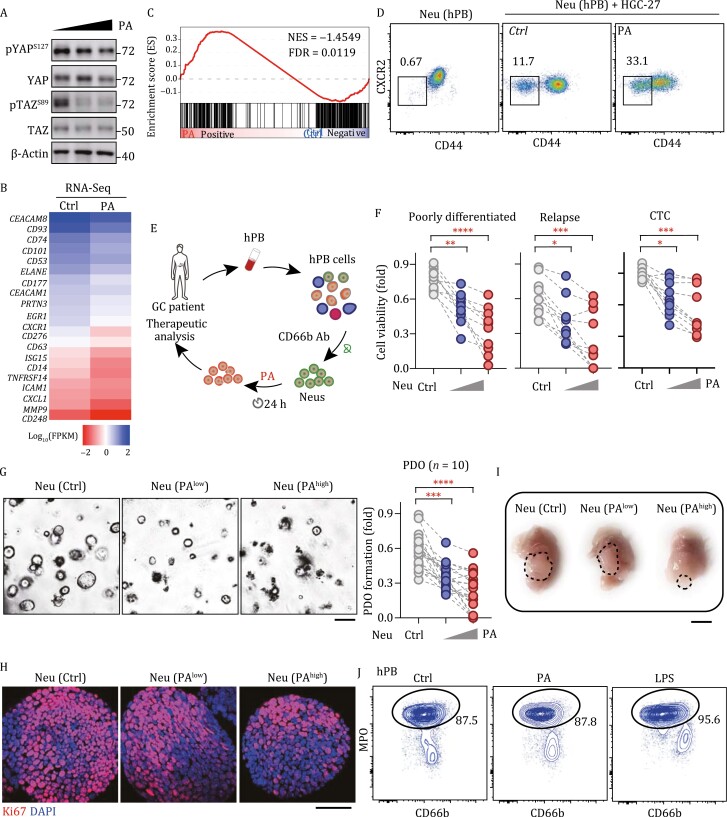
Therapeutic assessment of the Hippo-targeting strategy against human GC. (A) Relative amounts of tsNeus in neutrophils isolated from the peripheral blood of GC patient and cocultured with HGC-27 cells and treated with PA. (B) Immunoblotting analysis of the indicated proteins in PA-treated human tsNeus in panel (A). (C) Heat map of RNA-Seq-derived mRNA levels of indicated genes in PA-treated human tsNeus in panel (A). (D) GSEA of the RNA-Seq data in panel (C) for YAP signature genes identified from the aforementioned ChIP-Seq analysis. (E) Workflow of the mimicking of neutrophil therapy. CD66^+ ^cells were isolated from the peripheral blood of GC patient by performing FACS and were treated with PA for subsequent analysis. (F) *In vitro* killing assay of PA-treated human neutrophils in three groups of human GC cell types, namely poorly differentiated, relapsed, and circular tumor cell (CTC) types (*n* = 10). One-way ANOVA analysis with a Bonferroni post hoc test was performed. (G) Organoid formation of GC-patient-derived tumor cells cocultured with PA-treated neutrophils (*n* = 20). Representative microscopy images (left) and quantifications (right) are shown. Bar, 100 μm. Unpaired *t-*test was used for quantification analysis. (H) Ki67^+ ^staining of GC-patient-derived organoids cocultured with PA-treated neutrophils. Bar, 100 μm. (I) Orthotopic tumor formation of GC-patient-derived CTCs in nude mice receiving neutrophils isolated from the peripheral blood of GC patient and treated with PA (*n* = 5). (J) Expression of MPO in neutrophils isolated from the peripheral blood of GC patient and treated with PA or LPS. See also Fig. S7.

To further evaluate the therapeutic effect of the Hippo-YAP-targeting strategy, we isolated CD45^+^CD11b^+^Ly6G^+ ^neutrophils from the peripheral blood of GC patients. After incubating these patient-derived neutrophils with cells of the human GC cell line HGC-27, the percentage of tsNeus in them significantly increased (Fig. 7A). PA treatment further increased the fractions of tsNeus in the patient-derived neutrophils stimulated with HGC-27 (Fig. 7A). Meanwhile, the phosphorylation levels of YAP(S127)/TAZ(S89) were reduced (Fig. 7B). Moreover, our RNA sequencing analyses identified totals of 14,022 and 15,132 transcripts in the control and PA-treated human tsNeus, of which 797 and 562 genes exhibited significant up- and downregulations, respectively, in the PA-treated group relative to the control. Overall, PA-treated tsNeus displayed an upregulated transcriptional profile of neutrophil activation signature genes such as *CD14*, *CD54* (*ICAM1*), *TNFRSF14*, and *EGR1* (Fig. 7C). Subsequent GSEA also revealed a significant positive enrichment of YAP target genes defined by the aforementioned ChIP-Seq assay (Fig. 7D).

To mimic a neutrophil therapy, we first collected peripheral blood from GC patients to extract neutrophils and then treated these patient-derived neutrophils with PA for 24 h, and subsequently incubated them with isolated tumor cells from the GC patients (Fig. 7E). Consistent with our findings in mice, PA treatment strongly increased the killing ability of the patient-derived neutrophils toward the patient-derived GC cells including those from the poorly differentiated or relapsed cases (Fig. 7F). Importantly, PA also showed a strong therapeutic effect toward circulating tumor cells (Fig. 7F). These results were further confirmed by the observations in both a patient-derived organoid model and orthotopic nude mice xenograft model showing that PA in a dose-dependent manner inhibited organoid formation (Fig. 7G), proliferation (Figs. 7H and S7A), and tumor growth (Figs. 7I and S7I).

Finally, we also assessed the selectivity of PA activation of neutrophils. To this end, we treated neutrophils from the peripheral blood of GC patients with PA or LPS. We found that LPS but not PA highly increased the expression of myeloperoxidase (MPO) (Fig. 7J), an enzyme well studied for antimicrobial-defense-related inflammation (Liew and Kubes, 2019). Consistent with the effect of PA on expressions of inflammatory cytokines and *Elane* observed in mouse neutrophils (Fig. 6G and 6H), this result in human neutrophils further verified the selectivity and specificity of the immunotherapeutic strategy based on rational manipulation of the Hippo-YAP signaling in neutrophils.

## Discussion

Neutrophils remain a class of poorly understood components of the tumor microenvironment; in particular, the relationship between TANs and tumor progression is poorly understood (Zilionis et al., 2019; Veglia et al., 2021). The lack of a comprehensive understanding of TANs has thwarted efforts at developing any efficient neutrophil-based antitumor therapy. In the current work, we have provided a roadmap of TANs in mouse GC and human GC, with this roadmap defining CD44^−^CXCR2^− ^neutrophils as tumor-specific neutrophils (tsNeus). Importantly, CD54^+ ^tsNeu subsets were found to be strongly associated with GC progression and patient prognosis. We further dissected the transcriptional wiring of tsNeus, based on which we showed a proof of concept that a strategy involving the targeting of Hippo-YAP pathway may selectively activate neutrophils to kill tumor cells derived from patients with refractory GC.

Our current study revealed a lack of expression of CD44 and CXCR2 by tsNeus in GC, offering a way for both identifying and physically isolating these cells. In infected tissue or tumor microenvironment, the recruitment of neutrophils involves sequential steps including tethering, rolling, adhesion, crawling, and, finally, transmigration (Kolaczkowska and Kubes, 2013). Neutrophils usually remain CXCR2- before their maturation in bone marrow. Increased expression of CXCR2 triggers the release of bone marrow neutrophils into the circulation and promotes their subsequent recruitment to the tumor microenvironment (Eash et al., 2010). During this process, CD44 also plays an important role in neutrophil rolling and adhesion via its interaction with selectin expressed on the endothelium (McDonald et al., 2008). Once infiltrated into the tumor tissues, however, neutrophils appear to lose the expression of both CXCR2 and CD44, an event that might help neutrophils to stay in tumor tissues. Supporting this notion, we found peripheral blood neutrophils express high levels of CD44 and CXCR2 while tsNeus are CD44 and CXCR2 double negative. Consistent with our observations, a previous study also found a strong downregulation of CXCR2 in neutrophils infiltrated in pancreas cancer tissues (Evrard et al., 2018a).

We used CD54 in combination with CD101 as a gating strategy to separate and purify four subsets of tsNeus. In this regard, note that TANs have been observed to display an activated CD54^+ ^phenotype and can stimulate T cell responses at the earliest stages of lung cancer (Eruslanov et al., 2014). TANs have also been observed to display an activated CD54^+ ^phenotype in GC patients (Wang et al., 2017). Consistent with these studies, CD54^+ ^tsNeus, identified in our current work, showed higher killing ability than did other subpopulations. We found, in both mice and humans, that tsNeu expression of CD54 gradually increased with time in the early stages of GC, but then decreased with continued GC progression at advanced stages. The relationship between the dynamics of tsNeu subsets and tumor progression warrants further investigation.

The transcription factors involved at different stages of neutrophil development have been well characterized in steady and bacterial infection states (Yamanaka et al., 1997; Hock et al., 2003; Borregaard, 2010). Here, we showed YAP/TAZ-CD54 axis to be essential for the specification and antitumor function of tsNeus. We found the relative quantity of neutrophils in bone marrow, spleen or peripheral blood of *YAP*^*flox*/*flox*^*LyzM*^*cre*^ mice did not differ from those for wild-type control mice either in the steady state or upon tumor engraftment. However, deficiency of YAP/TAZ significantly impaired the generation and tumor infiltration of tsNeus, therefore, accelerating tumor progression. In contrast, treatment with PA, a compound targeting the upstream kinase LATS1/2 resulted in YAP/TAZ activation and enhanced the antitumor activity of tsNeus. Indeed, neutrophils in the tumor tissue showed the highest protein levels of YAP/TAZ but reduced expression levels of LATS1/2 compared to those from other tissues. Moreover, treatment with SHAP (Tang et al., 2020), a peptide-enhancing MST1/2 activity inhibited the tumor-killing ability of neutrophils (data not shown). These results indicated that the regulation of YAP/TAZ activity in neutrophils most likely depends on the classical Hippo-YAP pathway. As one of the limitations of our current study, the specific mechanism regulating Hippo-YAP for tsNeu differentiation remains largely unknown. In this regard, deletion of the upstream kinases MST1/2 or LATS1/2, which would activate YAP/TAZ to boost tsNeus may help to further clarify the functional role and regulatory mode of Hippo-YAP in neutrophils. The MST1/2 inhibitor XMU-MP-1 could also contribute to addressing these issues (Fan et al., 2016). Meanwhile, other factors such as inflammatory signals and metabolic cues in the tumor microenvironment should be considered for context-specific regulation of Hippo-YAP in neutrophils.

Due to the complex roles of neutrophils in antimicrobial defense, inflammation, and tumorigenesis, indiscriminately activating neutrophils may generate unwanted side effects such as damaging healthy tissue, and may even threaten the life of the patient. Thus, selective activation is key for neutrophil immunotherapy. In our current work, we designed a therapeutic strategy by using PA to pharmacologically intervene in the Hippo-YAP pathway. This strategy elicited a strong antitumor effect in both mouse GC and human GC, opening the possibility that cancer patients might benefit from receiving PA-treated neutrophils as an immunotherapy. To assess the selectivity of this strategy in the activation of neutrophils, we performed a series of experiments involving treating neutrophils with PA. This treatment induced gene expression in a pattern distinct from that of LPS signaling. Treating neutrophils with LPS strongly stimulated the expression of inflammatory cytokines including Tnfa, Il1b, Il6, and Il10, whereas PA had little effect in this regard. Instead, PA highly increased the expression of Elane to selectively kill cancer cells. Importantly, unlike LPS, PA did not activate neutrophils in the absence of tumor cells. Thus, targeting the Hippo-YAP pathway can selectively activate neutrophils to kill tumor cells, avoiding damaging normal tissues.

Given the reported protumor roles of neutrophils in tumorigenesis, it is thought that some neutrophils could act as polymorphonuclear myeloid-derived suppressor cells (PMN-MDSCs). The term “PMN-MDSCs” was originally used to refer to mouse model and cancer patient cells displaying immunosuppressive properties and that differentiated and expanded from immature granulocytes (Ostrand-Rosenberg and Sinha, 2009). Due to the lack of clear markers to distinguish PMN-MDSCs from granulocytes, the study of PMN-MDSCs in tumor progression has been challenging, even confusing. In some tumor models, CD115 was recognized as a useful PMN-MDSC marker (Youn et al., 2012). In this regard, we found that 3.8% of the tiNeus were positive for CD115 at day 10 of a xenograft GC model. This observation suggested that few tiNeus are PMN-MDSCs in the early stage of tumorigenesis, consistent with the overall antitumor activity of tsNeus. Meanwhile, most tsNeus isolated from GC patients appeared to be negative for CD115, indicating a separation of tsNeus from MDSCs. That said, we could not rule out the possibility that some tsNeus may behave like MDSCs in certain stages of tumor progression.

Interestingly, our study also identified a population of silent neutrophils (sNeus) expressing early lineage markers (CD34^+^c-Kit^+^) and having the potential to differentiate into tsNeus, a phenotype reminiscent of reserved stem cells. In this regard, according to a preview report, CD34^+^c-Kit^+ ^early neutrophils with protumoral activity were indicated to be accumulated in a murine tumor model (Zhu et al., 2018). The lineage specification of sNeus, as well as their ability to differentiate into tsNeus, remains to be clarified. It is also unclear whether the Hippo-YAP signaling regulates the potential stemness of sNeus and their differentiation into tsNeus.

In summary, our study characterized tumor-specific neutrophils in GC from the perspectives of cellular heterogeneity, molecular mechanism, and clinical relevance, paving the way to develop neutrophil immunotherapy against cancer. Also, we have highlighted a new type of immunotherapeutic approach, one involving targeting the Hippo-YAP pathway to selectively activate neutrophils for killing tumor cells without generating excessive amounts of inflammation.

## Methods

### Cells

MFC cells were obtained from the cell line resource of National Infrastructure (Beijing, China) and were grown in RPM I1640 medium (Invitrogen/Thermo Fisher Scientific, MA, USA), maintained in culture supplemented with 10% heat-inactivated FBS (Biological Industries), and 1% penicillin/streptomycin (Gibco/Thermo) at 37°C with 5% CO_2_ in a humidified incubator (Thermo Fisher Scientific). Cells were passaged for ≤3 months from the frozen early-passage stocks that had been received from the indicated sources. During the study, all cell cultures were periodically tested for mycoplasma using MycoAlert™ Mycoplasma Detection Kits (Lonza, ME, USA).

### Murine strains

*Yap1* ^*Floxed*^; *Taz*^*Floxed*^ mice were generated and characterized as described previously (Tang et al., 2020). Genomic DNA extracted from tail biopsies of these mice was used to evaluate offspring genotype. *LysM*^*cre*^ mice were purchased from the Jackson laboratory. *LysM*^*cre*^ mice were crossed with *Yap1*^*Floxed*^;*Taz*^*Floxed*^ mice to generate *Yap1*^*Floxed*^;*Taz*^*Floxed*^;*LysM*^*cre*/*cre*^ (*Yap1*;*Taz*^*DKO*^) mice. Successful deletion of YAP/TAZ in neutrophils was confirmed using immunoblotting. *Cd45.1* mice were kindly provided by Prof. Xiaolong Liu (SIBCB, China). C57BL/6J mice (male and female) used in this study were obtained each at an age of 6 weeks from SLAC Laboratory Animal (Shanghai, China).

All mice were housed under specific pathogen-free conditions in automated watered and ventilated cages on a 12-hour light/dark cycle and handled in accordance with the guidelines of the Institutional Animal Care and Use Committee of the Institute of Biochemistry and Cell Biology. The approval ID for the use of animals was SIBCB-NAF-14-004-S329-023 issued by the Animal Core Facility of SIBCB.

For subcutaneous murine GC models, MFC cells (1 × 10^6^ cells) were injected subcutaneously into the flank of the mice in a volume of 100 μL of RPMI 1640 medium. Tumor size was determined by taking caliper measurements of tumor length, width, and depth, and tumor volume was calculated as volume = 0.5236 × length × width × depth (mm^3^). No blinding was used in the treatment schedules for these experiments since the different treatments were identified by different marks on the tail. We used groups of 6–10 animals; based on our previous experience, such group sizes provide statistically significant data while keeping the number of animals used to a minimum.

### Preparation of murine tissues

All murine tissues in this study were prepared from mice sacrificed at the same time. BM cells were obtained from the sacrificed mice by flushing their femurs using a 21-gauge needle with RPMI1640 medium containing 1% FBS. For the isolation of SP cells, the whole spleen was dissected and homogenized into single-cell suspensions using a 1-mL syringe plunger with a 21-gauge needle. For the isolation of PB cells, a volume of 600–800 μL of blood was collected by performing retro-orbital bleeding. For the isolation of GC cells, xenograft tumor tissues (300–1000 mm^3^) were dissected into 1–2 mm pieces with scissors in PBS buffer and digested in RPMI 1640 medium with 1 mg/mL collagenase II (Sigma) and 100 μg/mL DNase I (Sigma) at 37°C for 30 min.

Subsequently, BM, SP, PB, and GC cells were filtered with a 70-μm cell strainer (BD Biosciences, San Jose, CA). After subjecting the cell culture to 5-minute centrifugation at 500 ×*g*, cells were resuspended with red blood cell lysis buffer (RT122-02, TIANGEN, Shanghai, China) to deplete red blood cells. Subsequently, the cells were washed 2–3 times and kept in RPMI 1640 medium with 1% FBS and 2 mmol/L EDTA at 4°C before use.

### Preparation of human GC samples

GC tissues were dissected into 1–2 mm pieces with scissors in PBS buffer and digested in RPMI 1640 medium with 1 mg/mL collagenase II and 100 μg/mL DNase I at 37°C for 30 min. After the depletion of red blood cells, the remaining cells were washed three times and kept in RMPI 1640 medium with 1% FBS and 2 mmol/L EDTA at 4°C before use. GC patients included in the study provided written informed consent for the use of their specimens. The studies were performed in accordance with the Declaration of Helsinki and approved by the Huashan Hospital Institutional Review Board (HIRB), Fudan University (Approval No.2017-222).

### Flow cytometry and cell sorting

Antibodies were purchased from BD, Biolegend, eBioscience, and Thermo Fisher (Table S4). For the identification of preNit1/2 and Nit1/2 Neus, cells were blocked with antimouse antibodies against CD16/32 (HI30) for 20 min at 4°C. After the exclusion of cell doublets and dead cells, the remaining cells were stained with fluorophore-conjugated antimouse antibodies against CD45 (30-F11), CD11b (M1/70), Ly6G (1A8), Ly6C (AL-21), CD54 (YN1/1.7.4), and CD101 (Moushi101) or antihuman antibodies against CD45 (HI30), CD11b (M1/70), CD66b (G10F5), CD54 (HA58), and CD101 (BB27) for 30 min at 4°C. Flow cytometry acquisition was performed on a 4-laser BD LSR Fortessa^TM^ (BD) using FACSDiva software, and data were subsequently analyzed using FlowJo software (Tree Star).

For the sorting of murine and human cells, cells (2 × 10^7^/mL) were incubated with the indicated fluorophore-conjugated antibodies for 30 min at 4°C. After the exclusion of cell doublets and dead cells, single cells were purified from the remaining material by performing fluorescence-activated cell sorting (FACS) using a FACS Aria III cell sorter and Diva software (both BD Biosciences) to achieve >98% purity.

### Wright–Giemsa staining

tsNeu subsets (5 × 10^4^ cells) were coated onto slides, dried for 15 min, fixed in methanol, and then stained with the Wright–Giemsa staining solution (C0135, Beyotime, Wuhan, China) according to the manufacturer’s protocol. Images were acquired with an Olympus BX53, and the stained areas of the cells (*n* = 20) were quantified by using Fuji ImageJ software (National Institutes of Health).

### Neu killing assay

MFC cells were first plated at 3,000 cells/well in 96-well plates. Different tsNeu subsets were then cocultured with the tumor cells at a ratio of 3:1 (tsNeus:MFC). After 48 h, nonadherent cells were washed away with PBS to remove dead cells and debris. Then the number of surviving cells was counted using a luminescence detector (GloMax® 20/20, Promega), and the percentage of tumor cells that were killed was calculated. The killing ability of each neutrophil subset was calculated using the equation Killing ability = (Luc^Mock^- Luc^treated^) × 100/Luc^Mock^.

### Single-cell RNA (scRNA) sequencing

Designated cells were sorted in PBS containing 0.05% BSA following the 10× Genomics protocol. The cell preparation time before loading onto the 10× Chromium controller was <2 h. Cell viability analysis and counting were performed using a microscope and trypan blue, and samples with viabilities of >85% were used for sequencing. Libraries were constructed using the Single Cell 3’ Library Kit V2 (10× Genomics). Transcriptome profiles of individual cells were determined by performing droplet sequencing following the protocol described by 10× Genomics. Once prepared, indexed complementary DNA (cDNA) libraries were sequenced with paired-end reads by using an Illumina NovaSeq 6000 apparatus (Illumina). The scRNA-Seq results reported in this paper were deposited in the Gene Expression Omnibus (GEO) database under accession number GSE168537.

### ChIP-Seq

ChIP assays were performed by using the Cut&Tag assay kit (TD-903, Vazyme) to decipher the YAP chromatin-binding profile in neutrophils isolated from human blood samples. Briefly, about 1 × 10^5^ neutrophils were suspended with 100 μL of wash buffer and incubated for 1 hour with concanavalin A-coated magnetic beads to tether the cells onto the beads. The bead-bound cells were resuspended in 50 μL of antibody buffer with 1 μg of anti-YAP antibody (sc-101199, Santa Cruz) or IgG control antibody (ab171870, Abcam) at 4°C with slow rotation overnight. Subsequently, the bead-bound cells were treated with 50 μL of secondary antibody diluted with the wash buffer for another hour at RT. The treated cells were washed three times with the dig wash buffer to remove unbound antibodies, and then further incubated with the hyperactive Pa-Th5 transposase adaptor complex (TD-903, Vazyme) to digest host genome and obtain fragmented DNA. The resulting DNA was purified by subjecting it to phenol-chloroform-isoamyl alcohol extraction and ethanol precipitation.

The DNA libraries were amplified by mixing 15 μL DNA with 5 μL of a universal N5XX and uniquely barcoded N701 primer (TD-202, Vazyme) followed by subjecting the mixture to 15 cycles at 98°C for 10 s, 60°C for 5 s 72°C for 1 min, and hold at 4°C. To purify the PCR products, 1.2× volumes of VAHTS DNA Clean Beads (N411, Vazyme) were used and incubated at room temperature for 10 min. The size distribution of the libraries was examined by performing an Agilent 2100 analysis. Sequencing was performed using the Illumina HiSeq X-ten platform (Shanghai Biotechnology Co., China). Sequencing raw reads were preprocessed by filtering out sequencing adapters, short-fragment reads, and other low-quality reads. Bowtie (version 0.12.8) was then used to map the clean reads to the human hg19 reference genome. Peak detection was performed by using MACS2 (version). The protein-binding motifs were identified using HOMER software. The results of the ChIP-Seq analysis reported in this paper were deposited in the GEO database with accession number GSE188962.

### Adoptive cell transfer

Cd45.1 recipient mice and Cd45.2 donor mice were subcutaneously injected with MFC cells (2 × 10^6^) to induce tumor formation. After tumors were allowed to grow for 5 days, Cd45.2 tsNeu1/2 (1 × 10^5^ cells) or Cd45.2 tsNeu3/4 (1 × 10^5^ cells) was sorted and resuspended in 20 μL of RPMI 1640 medium, and then transferred intratumorally into Cd45.1^+ ^wild-type recipients. At 48 h after cell transfer was conducted, tumors from Cd45.1 mice were collected and analyzed using flow cytometry.

### Immunoblotting

Neutrophils were lysed with 20 mmol/L Tris·HCl, pH 8.0, 100 mmol/L NaCl, 0.5% Triton X-100, 1 mmol/L EDTA, and 1 % protease inhibitor cocktail tablets (Roche) on ice for 30 min. Cell lysates were then boiled with 5× SDS loading buffer. The proteins were separated using SDS-PAGE, transferred to a PVDF membrane (Bio-Rad) and further incubated with antibodies. The antibodies used for immunoblotting targeted YAP (sc-101199, Santa Cruz, 1:500), pYAP^S127^ (13008, Cell Signaling Technology, 1:500), TAZ (22439, Abcam, 1:500), pTAZ^S89^ (59971S, Cell Signaling Technology, 1:500), β-actin (A2228, Sigma-Aldrich, 1:5,000). Raw images and corresponding quantifications are shown in Table S6.

### Real-time PCR

Total RNA was isolated from cells by using Trizol reagent (Vazyme Biotech, Nanjing, China) according to the manufacturer’s instructions. The isolated RNA was reverse transcribed into cDNA using HiScript II (Vazyme Biotech). Quantitative real-time PCR was performed with a CFX96™ real-time PCR system (Bio-Rad, Hercules, CA) and SYBR Green PCR master mix (Yeasen, Shanghai, China). The fold change in the gene expression was calculated by using the comparative Ct method, and three replicates of each cDNA sample were tested. The sequences of the primers are listed in Table S7.

### Statistical analysis

Both cellular and animal studies have tended to be underpowered. Estimations of sample sizes for planned comparisons of two independent means using a two-tailed test were undertaken using the SAS statistical software package (9.1.3). Data are presented as mean ± SD for continuous variables and as frequencies and proportions for categorical variables. Continuous data were compared using Student’s *t*-tests (comparing two variables) or one-way ANOVA analysis (comparing multiple variables). Survival curves for mice were calculated according to the Kaplan–Meier method; survival analysis was performed using the log-rank test. A value of *P* < 0.05 was considered to indicate a significant difference. Three biological replicates were used throughout the study, except for scRNA-Seq and immunoblotting for which only two biological replicates were used.

## Supplementary information

The online version contains supplementary material available at https://doi.org/10.1093/procel/pwac045.

pwac045_suppl_Supplementary_Materials

pwac045_suppl_Supplementary_Tables

## Data Availability

Single Cell RNA sequencing raw data have been deposited in the Gene Expression Omnibus database under the accession no. GSE168537. The GEO accession numbers for the ChIP-Seq reported in this paper are GSE188962. This study did not generate new unique reagents.

## Abbreviations

BM: bone marrow
ChIP: chromatin immunoprecipitation
DN: CD54^−^CD101^−^
DP: CD54^+^CD101^+^
eNeP: early unipotent neutrophil progenitor
GC: gastric cancer
GFI-1: growth factor independent 1
GMPs: granulocyte monocytes progenitors
GSEA: gene set enrichment analysis
*H. pylori*: *Helicobacter Pylori*
immNeu: immature neutrophil
mNeu: mature neutrophil
MPO: myeloperoxidase
PA: phosphatidic acid
PB: peripheral blood
PMN-MDSCs: polymorphonuclear myeloid-derived suppressor cells
preNeu: neutrophil precursor
proNeu: neutrophil progenitor
PT: peritumoral tissue
ROS: reactive oxygen species
SCENIC: single-cell regulatory network inference and clustering
scRNA-Seq: single cell RNA sequencing
sNeu: silent neutrophil
SP: spleen
SP1: CD54^−^CD101^+^
SP2: CD54^+^CD101^−^
TANs: tumor-associated neutrophils
tiNeus: tumor-infiltrated neutrophils
t-SNE: t-distributed stochastic neighbor embedding
tsNeus: tumor-specific neutrophils
TSSs: transcription start sites
UMAP: uniform manifold approximation and projection
