# Supplementary material for: A YAP/TAZ-CD54 axis is required for CXCR2−CD44− tumor-specific neutrophils to suppress gastric cancer
Source: Protein Cell. 2022 Oct 26;14(7):515–33. doi: 10.1093/procel/pwac045 (PMC10305741; doi:10.1093/procel/pwac045)
Supplement: pwac045_suppl_Supplementary_Materials [file pwac045_suppl_supplementary_materials.pdf]

Supplemental materials

Figure S1~S7.

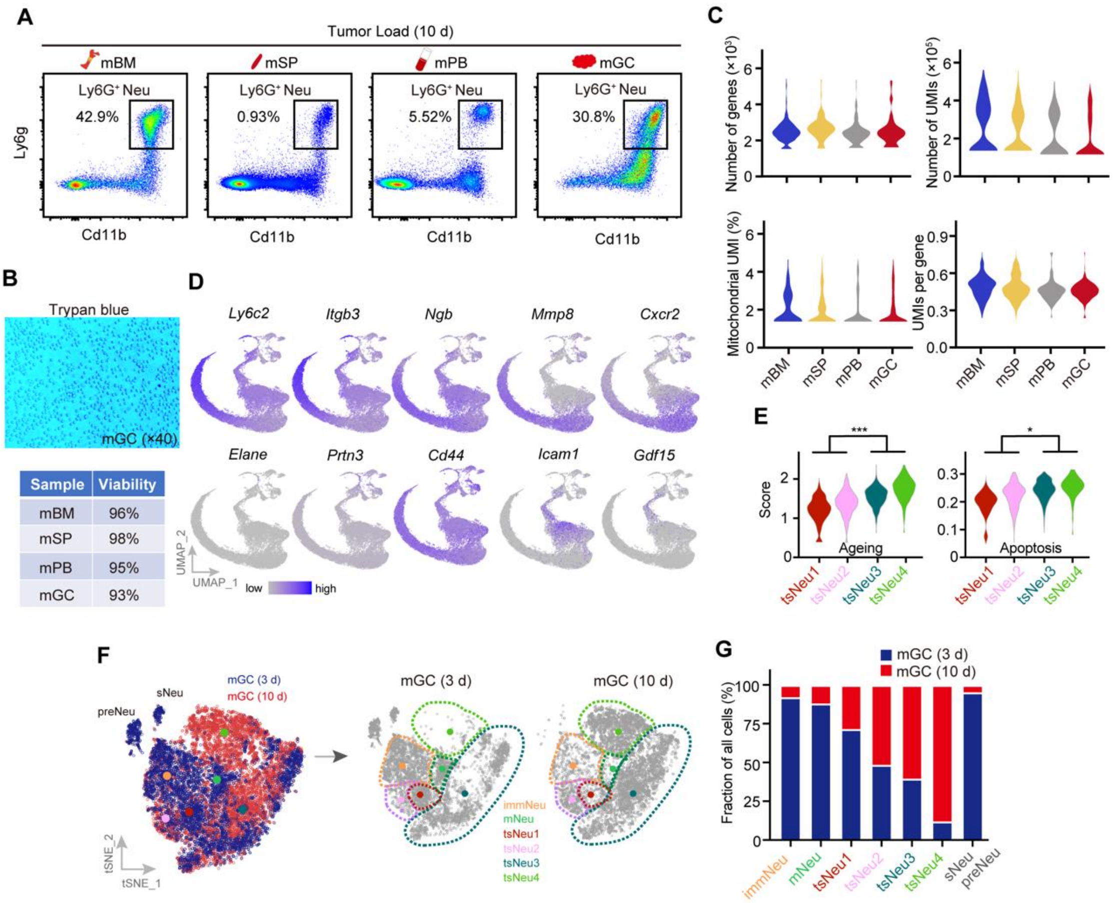

Figure S1 Neutrophils display transcriptomic heterogeneity. (A) Results of sorting of BM-, SP-, PB- and GC Ly6G-positive neutrophils (Neus) sorted from three tumor-bearing mice according to the described gating strategy. (B) Viability levels of neutrophils from panel (A) were measured by Trypan blue staining and the neutrophils were then loaded onto a 10×Chromium single-cell controller. (C) Violin plots of the number of genes, number of UMIs, mitochondrial UMI percentage, and UMIs per gene of all QC-passed cells in the indicated samples. (D) Transcriptional patterns of the indicated genes projected on their respective UMAP plots. (E) Violin plots of aging score and apoptosis score for the indicated clusters. (F) t-SNE plots of $CD11b^{+}Ly6G^{+}$ neutrophils in a murine subcutaneous GC model at the indicated time after the mice were injected with MFC cells. (G) Bar graph showing the relative populations of the indicated subsets from mice bearing tumors for 3 days or 10 days.

Related to Figure 1.

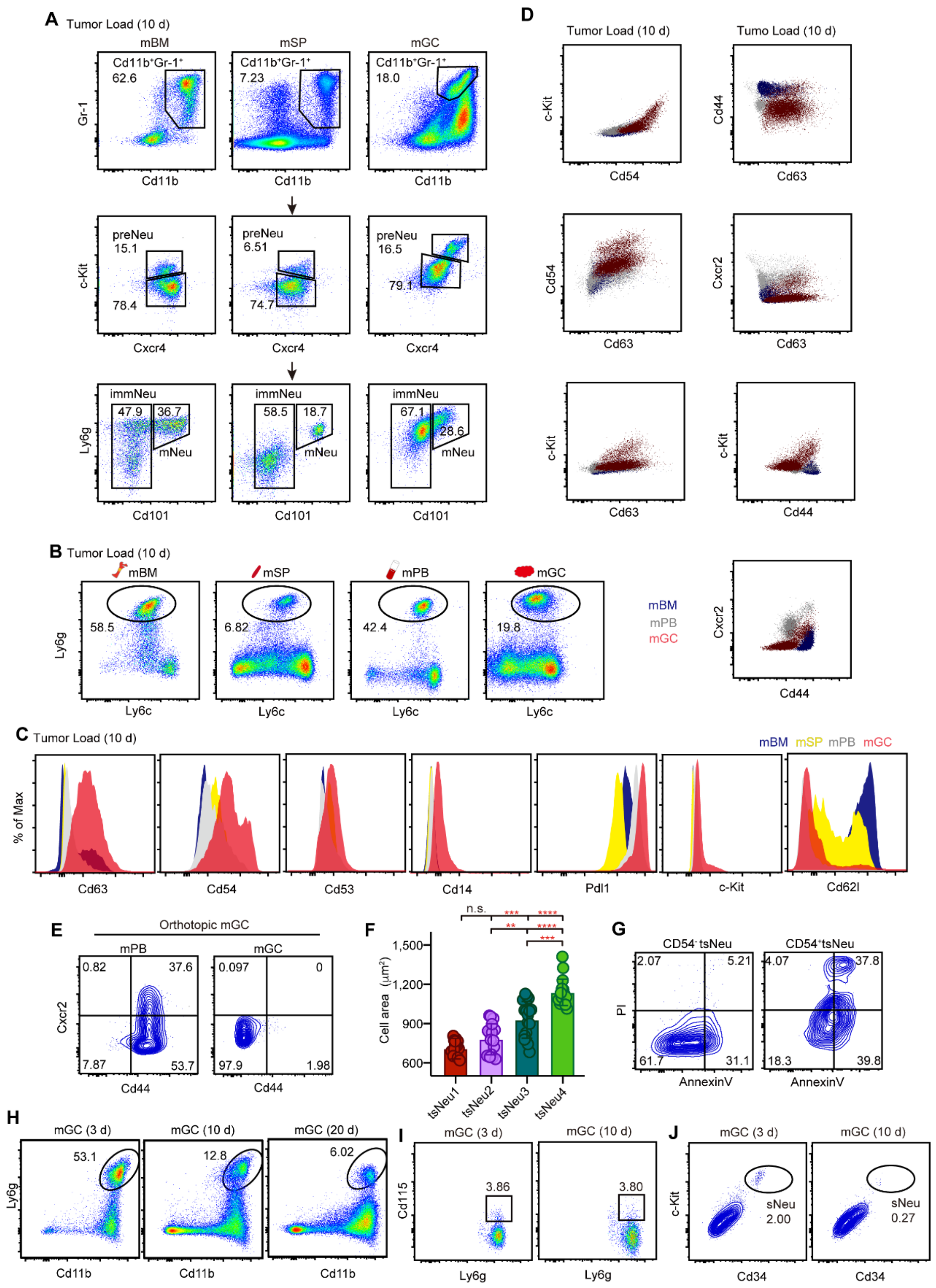

Figure S2 Neutrophils display morphological and functional heterogeneity. (A) Representative FACS plots of preNeus, immNeus and mNeus from tumor-bearing mice according to the indicated gating strategy. (B) Representative FACS plots of total number of neutrophils ($Ly6G^{+}Ly6C^{lo}$) in BM, SP, PB and GC tissue from tumor-bearing mice. (C) Histograms of the indicated surface markers in neutrophils from the indicated samples. (D) Plots showing the differential expression of the indicated markers. (E) Flow cytometry plots

of tsNeus from peripheral blood (mPB) and GC tumors (mGC). Mice were injected with MFC cells for 10 days. (F) Cell surface areas of sorted tsNeu subpopulations. The stained areas of the cells were quantified by using Fuji ImageJ software (n=20). (G) Apoptosis results for sorted $CD54^-$ tsNeus and $CD54^+$ tsNeus, respectively. (H) FACS plots of tiNeus ($CD11b^+Ly6G^+$) in a murine subcutaneous GC model at the indicated times after the mice were injected with MFC cells. (I) Expression of CD115 in Ly6G+ cells. (J) Representative FACS plots of sNeus from the indicated samples.

Related to Figure 2.

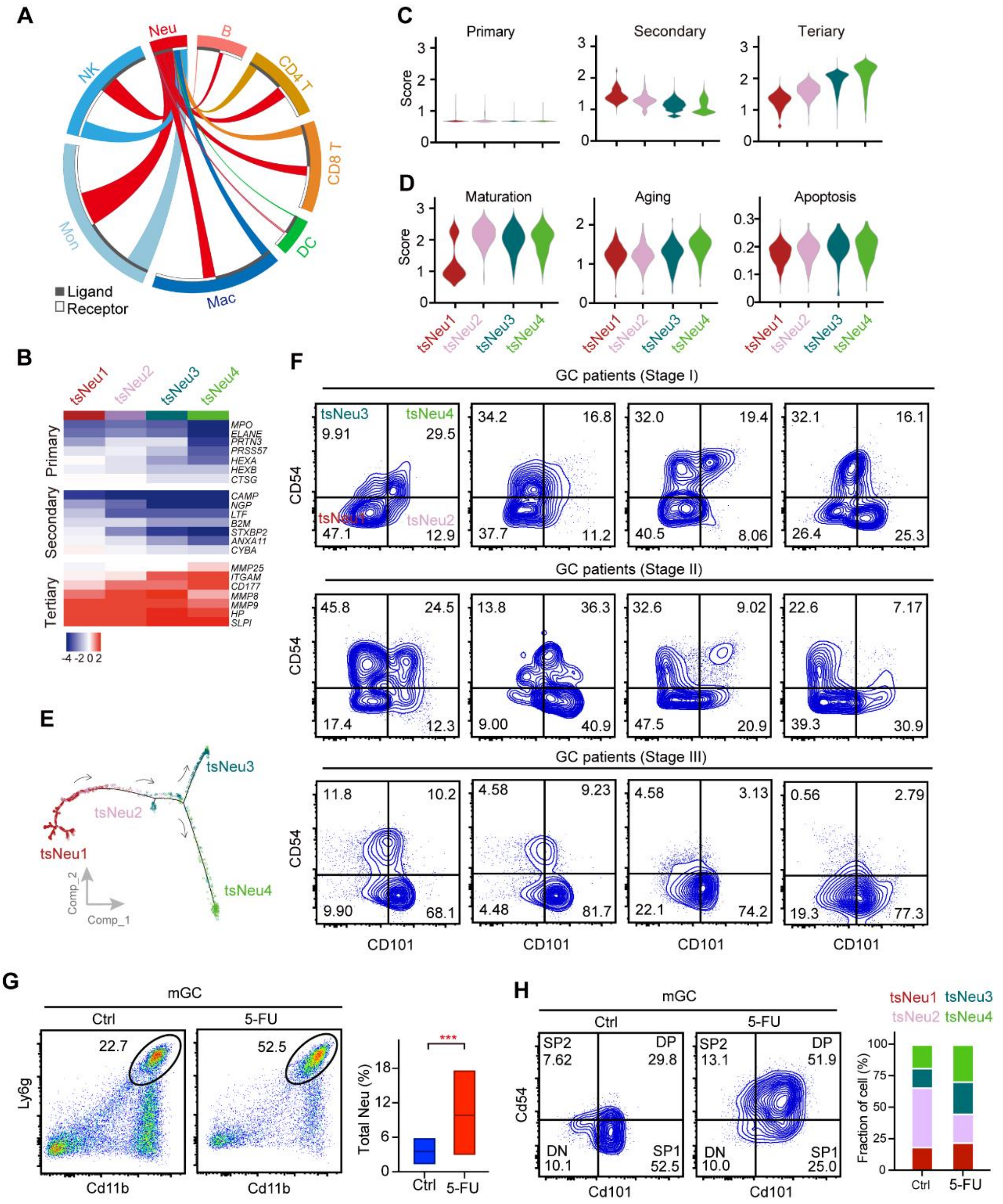

Figure S3 scRNA-Seq analysis of human GC samples characterizes four tsNeu subsets. (A) Circus plot showing ligand-receptor interactions between Neu and other immune cell types. (B) Heatmap showing the expression levels of selected functionally relevant genes. (C,D) Violin plots of primary (azurophil), secondary (specific), tertiary (gelatinase score and secretory), maturation, aging, and apoptosis scores for the Neu clusters. (E) Trajectory prediction for the tsNeu subsets. (F) Representative flow cytometry analysis plots showing relative amounts of tsNeu subsets in GC patients with indicated tumor stages. (G,H) Total Neu and tsNeu subpopulations in the murine GC tissue from mice receiving 5-FU treatment. Related to Figure 3.

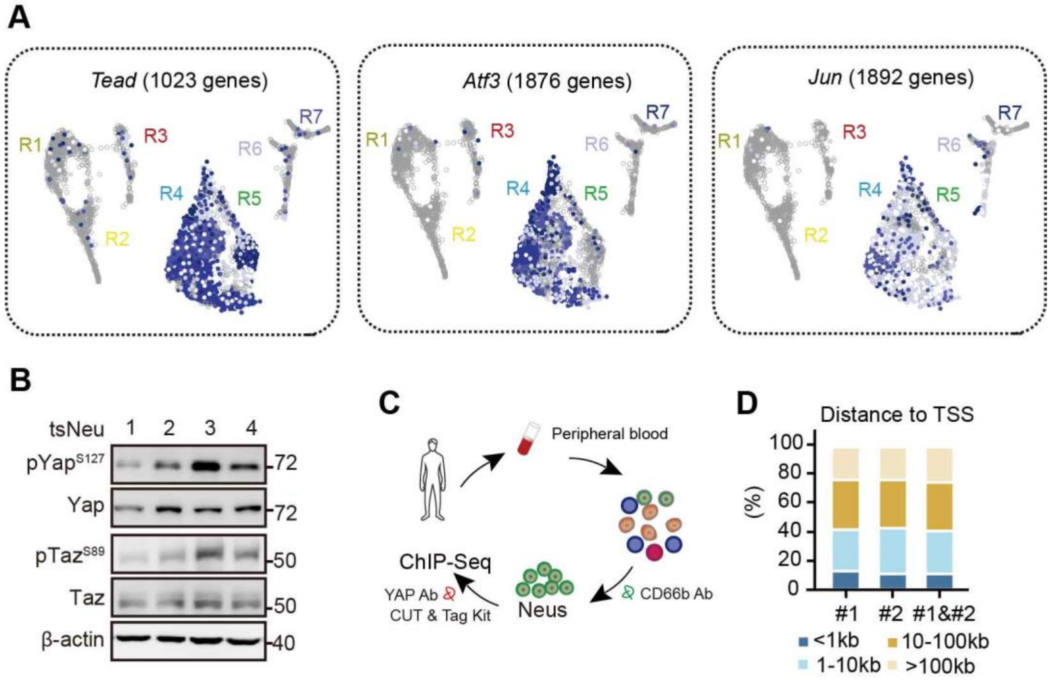

Figure S4 Characterization of the Hippo regulon in neutrophils. (A) Activities of the three identified tsNeu-associated regulons, namely *Teads*, *Arf3* and *Jun*, projected on their respective UMAP plots. (B) Immunoblotting analysis showing the expression levels of $pYAP^{S127}$, $pTAZ^{S89}$, YAP and TAZ in the four indicated tsNeu subsets. (C) Chip-Seq analysis workflow. ChIP assays were performed with anti-YAP antibodies from Abcam (#1, ab39361) and Santa Cruz (#2, sc-A2319) in human $CD66^{+}$ Neus, followed by carrying out the ChIP-Seq analysis. (D) Absolute distances of YAP #1 peaks, YAP #2 peaks, and overlapping YAP #1/#2 peaks each to the nearest TSS.

Related to Figure 4.

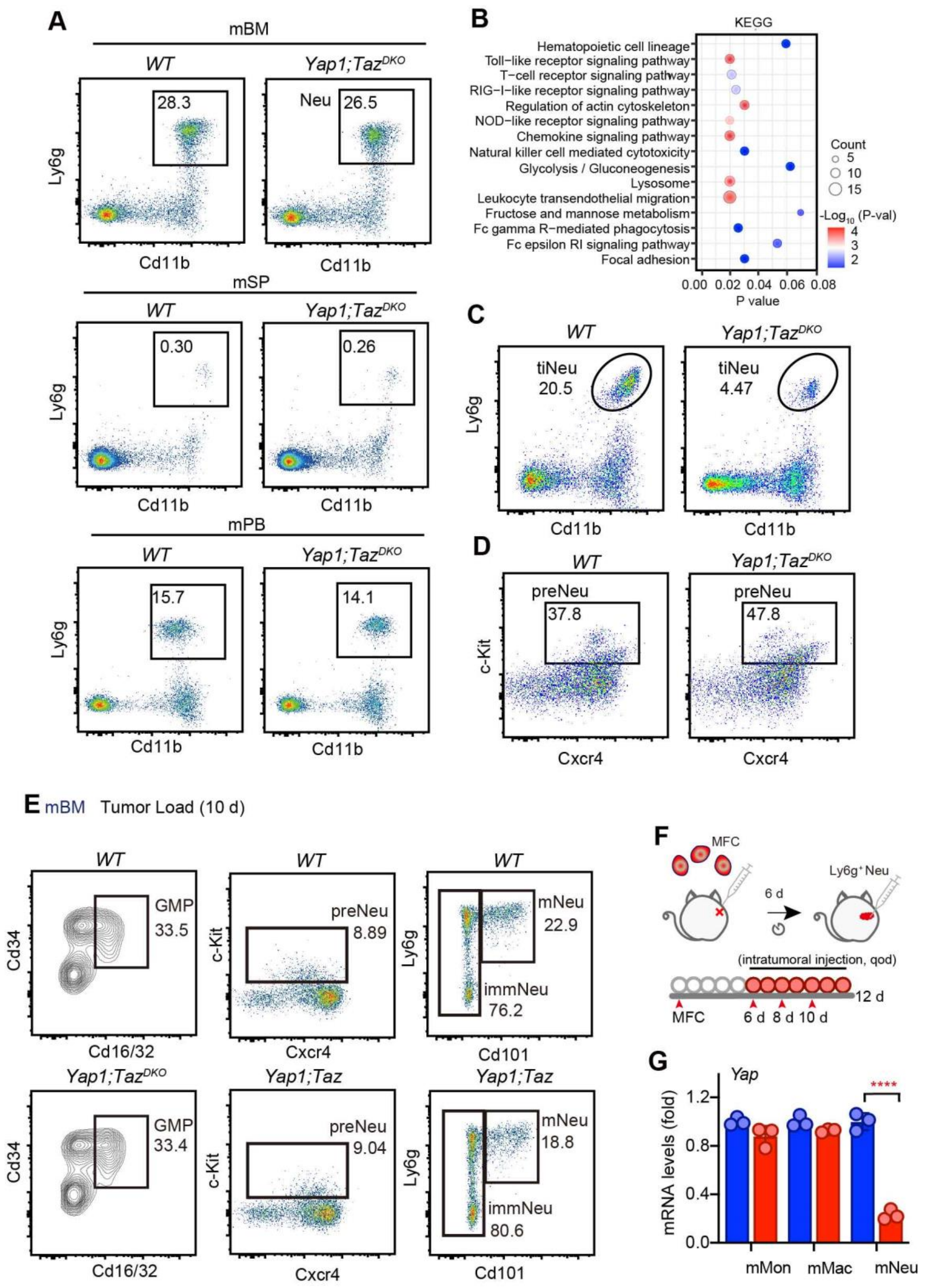

Figure S5 The different effects of YAP/TAZ deficiency in mice with and without tumor Load. (A) FACS plots of CD11b$^+$Ly6G$^+$ neutrophils in the indicated tissues from the *Yap1$^{flox/flox}$Taz$^{flox/flox}$LyzM$^{cre/cre}$* (*Yap1;Taz^{DKO}*) mice and control mice. (B) KEGG analysis showing top 15 enriched pathways in the *Yap1$^{flox/flox}$Taz$^{flox/flox}$LyzM$^{cre/cre}$* mice versus control mice. (C) FACS plots of tiNeus (CD11b$^+$Ly6G$^+$) in the tumor tissues from the

Yap1$^{flox/flox}$*Taz*$^{flox/flox}$*LyzM*$^{cre/cre}$ (*Yap1;Taz*^{DKO}) mice and control mice. (D) FACS plots of preNeus in the tumor tissues from the tumor-bearing *Yap1*$^{flox/flox}$*Taz*$^{flox/flox}$*LyzM*$^{cre/cre}$ mice (*Yap1;Taz*^{DKO}) and control mice. (E) FACS plots of GMP (Lin^{-}c-Kit^{hi}CD11b^{-}CD34^{+}CD16/32^{+}), preNeus (Lin^{-}CD115^{-}Siglec^{-}c-Kit^{+}CXCR4^{+}), immNeus (Ly6G^{lo}CD101^{-}) and mNeus (Ly6G^{lo}CD101^{+}) gated from tumor-bearing *Yap1*$^{flox/flox}$*Taz*$^{flox/flox}$*LyzM*$^{cre/cre}$ mice (*Yap1;Taz*^{DKO}) and control mice. (F) Experimental workflow of the tumor formation assay after transferring tsNeu cells to tumor-bearing mice. (G) mRNA levels of *Yap* in the indicated murine cells.
Related to Figure 5.

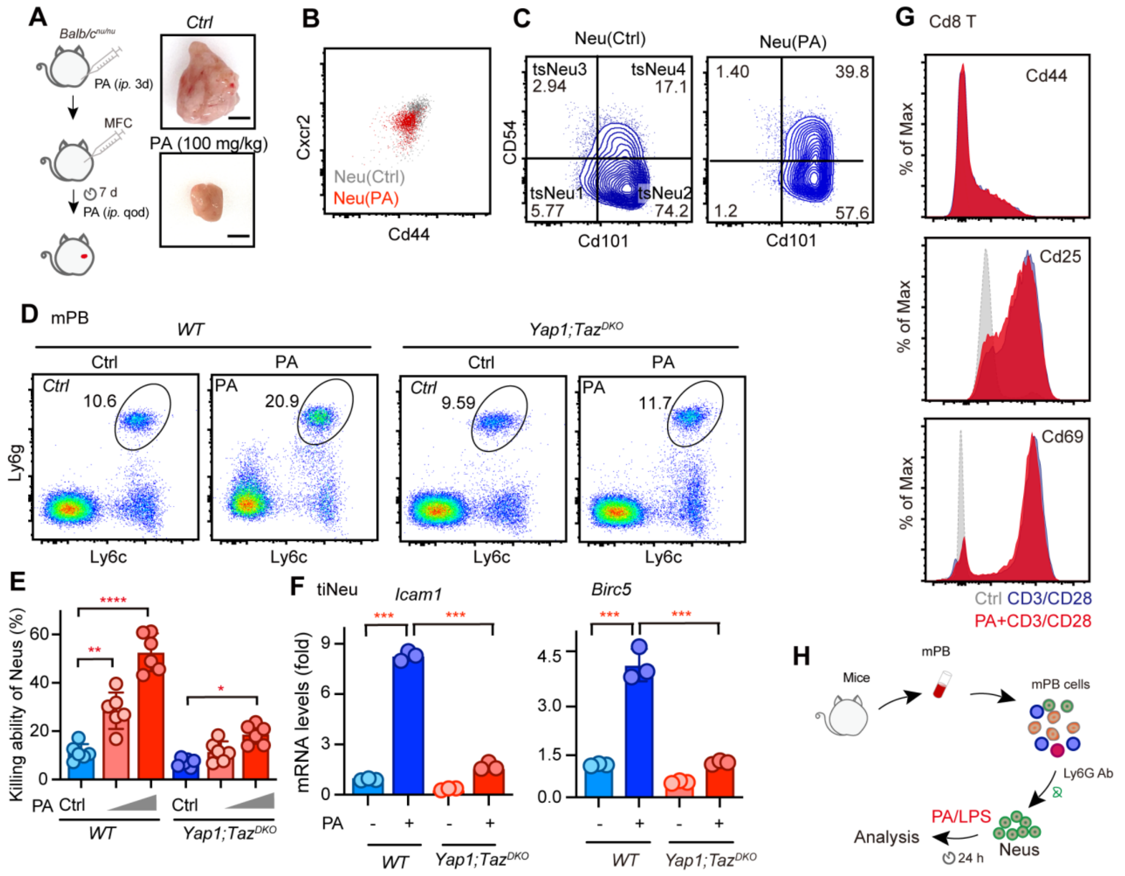

Figure S6 PA treatment redirects neutrophils for antitumor immunotherapy. (A) *In vivo* tumor formation in nude mice treated with PA (n = 6). Workflow of MFC-induced tumor formation assay (left). (B) Flow cytometry plot of peripheral blood $CD44^{-}CXCR2^{-}$ neutrophils treated with PA. (C) tsNeu subpopulations in PA-treated murine tumor tissues. (D) Flow cytometry plots of neutrophils in peripheral blood from wildtype (*WT*) and *Yap1$^{flox/flox}$Taz $^{flox/flox}$LyzM$^{cre/cre}$* (*Yap1;Taz^{DKO}*) mice treated with PA. (E) *In vitro* tumor-killing abilities of PA-treated wildtype and *Yap1;Taz^{DKO}* neutrophils after they were co-cultured with MFC cells. Bar graph showing the tumor-killing activities of wildtype or Yap/Taz-deficient neutrophils treated with PA (n =6). (F) mRNA levels of *Icam1* and *Birc5* in tiNeus from PA-treated mice. (G) CD3/CD28-induced activation of splenic $CD8^{+}$ T cells after mice were treated with PA for 3 days. (H) Workflow of PA/LPS treatment of neutrophils.

Related to Fig. 6

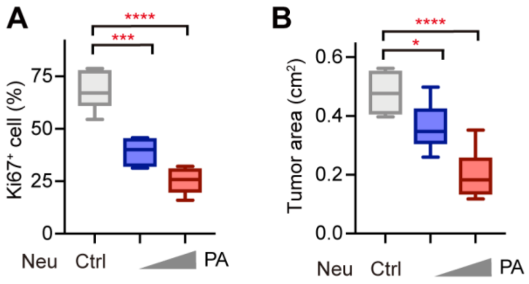

Figure S7 Assessment of the Hippo-targeting strategy for antitumor Immunotherapy. (A) Quantification of Ki67$^+$ staining in a GC patient organoid co-cultured with GC patient-derived neutrophils treated with PA. (B) Orthotopic tumor surface areas of in mice receiving PA-treated neutrophils (n = 5).

Related to Figure 7
